# Seed Oligomers Regulate Sequence Development through a Templating Effect in Simulated Irreversible Step-Growth Copolymerization

**DOI:** 10.1021/acs.macromol.5c01639

**Published:** 2025-12-05

**Authors:** Wenxin Xu, Nhu Q. Nguyen, Kateri H. DuBay

**Affiliations:** 1 Department of Chemistry, University of Virginia, Charlottesville, Virginia 22904, United States; 2 Fulbright University Vietnam, Ho Chi Minh City 700000, Vietnam

## Abstract

The sequences of copolymers determine their material properties, driving significant interest in realizing the control of synthetic copolymer sequences. Templating with preformed seeds provides one potential route to efficiently obtain sequence-controlled polymers. However, the complicated interplay between various factors during polymerization requires more study. We previously found that sufficient chain stiffness and interchain attractions could trigger an emergent self-templating effect through the nematic alignment of nascent oligomers. Herein, we probe this templating effect more directly by adding preformed seed chains in simulations of irreversible step-growth copolymerization. The results show that the final sequences are influenced by the addition of seed chains, which affect the emergent microphase separation and reaction kinetics. We also find that the final sequences vary with the sequence, length, and intrinsic stiffness of the preformed seeds, as well as with the solvent viscosity and the reaction barriers. This work provides insight into the factors influencing seed templating in step-growth copolymerization and highlights a potentially simple and effective route to improve sequence control through the addition of preformed seed chains.

## Graphical abstract

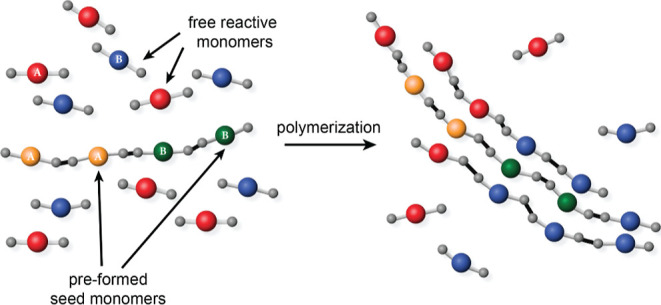

## 1 Introduction

The phase behavior and material properties of copolymers depend on the primary sequences of their monomers;^1–5^ different sequences of the same overall fractions of monomer components yield different self-assembled structures and macroscopic properties.^6^ Since different sequences enable a variety of advanced applications,^3,7–10^ significant efforts have been made to control or bias sequences in synthetic copolymerizations. Many synthetic approaches have been developed to do so, such as living radical polymerization^11–14^ for chain-growth copolymerization and multicomponent reactions^15,16^ for step-growth copolymerization. However, synthetic routes with multiple steps increase the difficulty of large-scale production of sequence-controlled or sequence-biased polymers, especially for step-growth copolymerization where bond formation is harder to control since all unbound polymer functional groups are reactive and the polymer sequence is not formed sequentially in time.

Another critical mechanism for sequence regulation is the templating effect, which is inspired by the reproduction of biopolymer sequences.^17^ For instance, the replication of DNA and RNA relies on template strands, to which free monomers are linked by hydrogen bonding during polymer bond formation.^18–20^ The sequence of the newly formed oligonucleotides is then determined by the templating polynucleotides with high sequence selectivity and regioselectivity.^21–23^ In recent years, this templating effect has been applied to make synthetic polymers,^24^ such as the irreversible 2D polymerization of polyaramid^25,26^ and the surface-initiated polymerization of conductive polymer brushes.^27^ Simulation work has also demonstrated the efficacy of a template surface in influencing polymer sequences within reversible polycondensation and chain-growth polymerization.^28,29^ In the production of supramolecular copolymers, the addition of template “seed” chains helps in obtaining the desired sequences of monomers with controlled lengths and dispersities, an effect known as the “seeding effect”.^30–32^

When possible, a templating approach with seed oligomers made of the same material as the final desired polymers can increase the purity of the final polymers while reducing overall cost.^33^ However, the efficiency of this type of seeded self-templating in polymer synthesis is impacted by a complex interplay of reactant properties and reaction conditions. As a result, it is necessary to improve our understanding of how such seeds influence the copolymerization process in order to develop simple synthetic routes for step-growth copolymerization.

In our previous work, we have found that certain conditions can trigger self-templating effects even in the absence of preformed seed oligomers.^34–38^ In these cases, an emergent microphase separation among the earliest forming oligomers can influence polymerization kinetics, resulting in biased sequences, even within one-pot step-growth copolymerization. This microphase separation behavior and biased sequence effect depend on relatively weak nonbonded attractions between monomer pairs,^34^ solvent selectivity,^36^ activation energy,^37^ and monomer diffusivity.^38^ Furthermore, if the chain persistent length, *l*_p_, is at least 10 monomers, then nascent oligomers can nematically align within the aggregates, impacting the resulting polymer sequence. A characteristic peak appears in the block-length distribution for these semistiff chains, suggesting a self-templating mechanism where chains replicate the sequences of the earliest oligomers.^35^

In this research, we build on our previous work by investigating the self-templating effects arising from the addition of preformed seed chains during step-growth copolymerizations at the irreversible limit and study the effect of these seed oligomers on the sequence and reaction kinetics. The model is described in Section 2. In Section 3.1, we determine the templating effects from preformed seed chains of various sequences and show that the seed oligomer sequence influences the final sequences and overall reaction kinetics. In Section 3.1, we investigate other factors influencing the seeding effect, including the chain length of the seeds and the block lengths within the seeds, the intrinsic stiffness of the chains, and the solvent viscosity and reaction kinetics. In Section 4, we conclude with the main observations and the potential impact of this seeding approach on sequence control during the step-growth copolymerization.

## 2 Methods

### 2.1 Coarse-Grained Model for a Mixture of Seeds and Monomers

In this work, we utilize a coarse-grained model for irreversible step-growth copolymerization that was previously developed in our group.^34^ Each monomer in this model is represented by three connected beads: one center bead (type-**1**) and two linking beads (type-**2**), describing monomer sections with different interactions (Figure 1a).

**Figure 1:**
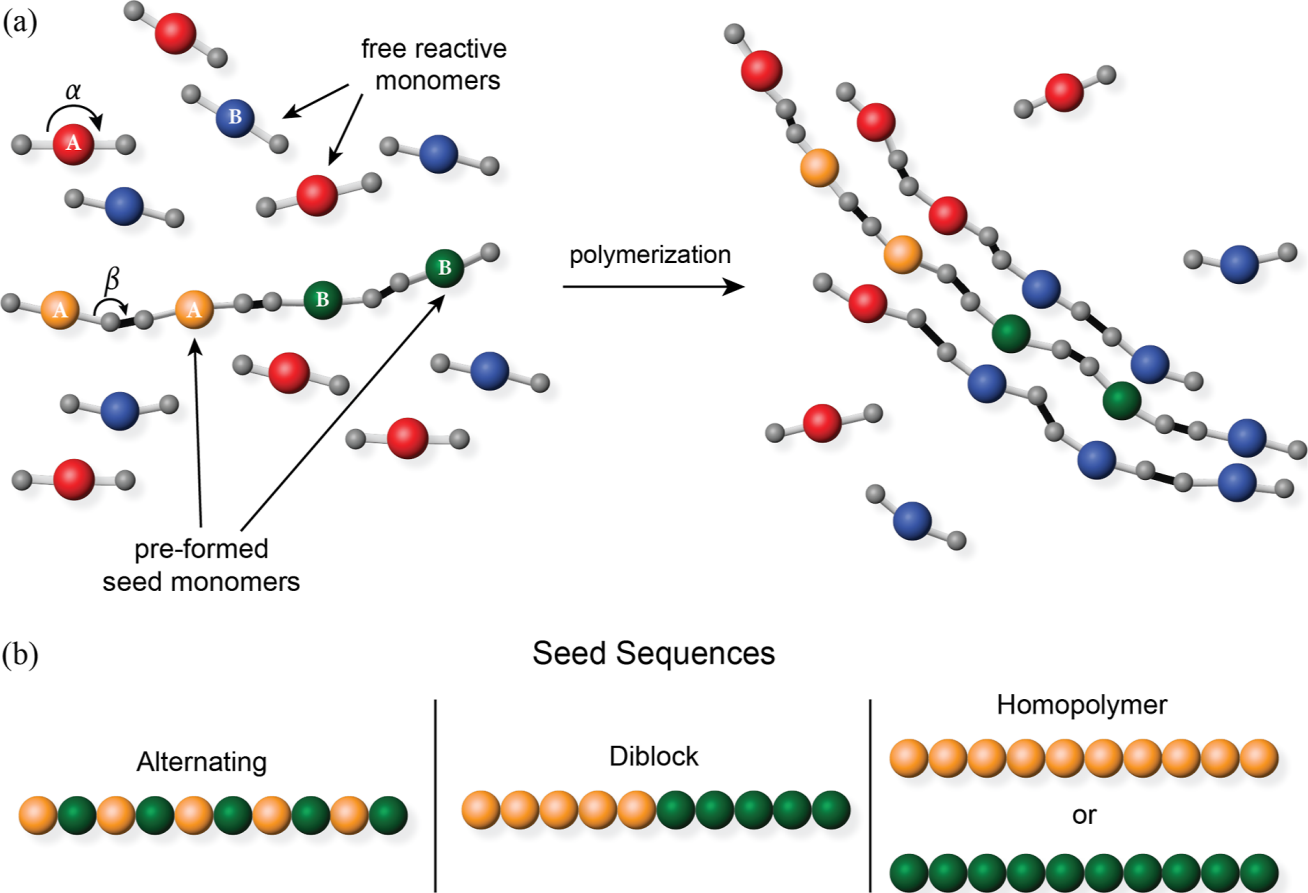
**Coarse-grained polymerization model.** (a) Schematic of monomers, chains, and polymerization. The system starts from a random mixture of monomers and preformed seed oligomers. Each monomer is composed of one central particle of type **1** and two linking particles of type **2** (gray particles). There are two types of type-**1** particles: **A** and **B**. To distinguish between free monomers and seed monomers, we show **A** monomers that start out unbound in red and **B** monomers that start out unbound in blue, and we use orange and green to represent **A** and **B** seed monomers, respectively. α and β refer to intramolecular and intermolecular angles. Type-**1** particles interact via nonbonded attractions and repulsions. The attraction between the same species, ε_AA_ and ε_BB_, is 1.0 *k*_B_ *T*, while ε_AB_ is 0 *k*_B_ *T*. The monomers in the seeds are linked by an irreversible intermolecular bond (black) between type-**2** particles. During the polymerization, additional intermolecular bonds can be formed between monomers by overcoming the activation energy. The monomers can either add to the ends of seeds or polymerize separately from the seeds. Polymerization proceeds until the reaction extent, *p*, reaches 90%. (b) Sequences of seeds. We examine the effects of three seed sequences: alternating, diblock, and homopolymer.

We use a modified Lennard-Jones (LJ) interaction potential (*E*_LJ_ in eq 1) between type-**1** particles, which introduces differences in the non-bonded intermolecular interactions between monomers:

1 $$E_{LJ}\left(1,1^{\prime }\right)=\{\begin{array}{ll} 4\varepsilon_{att\left(1,1^{\prime }\right)}\left[{\left(\frac{\sigma }{r_{\left(1,1^{\prime }\right)}}\right)}^{12}-{\left(\frac{\sigma }{r_{\left(1,1^{\prime }\right)}}\right)}^{6}\right], & r_{0}\leq r_{\left(1,1^{\prime }\right)}<2.5\sigma \\ 4\varepsilon_{rep\left(1,1^{\prime }\right)}\left[{\left(\frac{\sigma }{r_{\left(1,1^{\prime }\right)}}\right)}^{12}-{\left(\frac{\sigma }{r_{\left(1,1^{\prime }\right)}}\right)}^{6}\right]+C & r_{\left(1,1^{\prime }\right)}<r_{0} \end{array}$$

where σ is the size of type-**1** particles and *r*_0_ is the distance of the energy minimum which is equal to 2^1/6^σ. The constant *C* is chosen to ensure the continuity of the LJ potential at *r*_0_. The repulsive part of the LJ potential remains fixed (see Supporting Information), while the attractive portion is varied by modifying the constant ε_att_. Since the two kinds of type-**1** beads, **A** and **B**, correspond to two distinct monomer species, there are three constants—ε_AA_, ε_BB_, and ε_AB_—which represent the attractions between **AA**, **BB**, and **AB**, respectively. Unless otherwise stated, ε_AA,BB_ and ε_AB_ are set to 1.0 *k*_B_ *T* and 0 *k*_B_ *T*, respectively, as these interactions gave rise to the microphase separation observed in the previous study.^34^

We use a harmonic potential for all bonds and angles (see in Supporting Information). By adjusting the harmonic coefficient of the intramolecular angle (α in Figure 1a), chains with different persistence lengths, *l*_p_, are generated (see in Supporting Information).^35^ The intermolecular harmonic bonds, by which monomers in the seeds and growing oligomers are connected (Figure 1a), cannot be broken once formed, so the simulation represents the reaction at the limit of irreversible polymerization. All bond and angle potentials are the same for seeds and oligomers formed during the simulation.

### 2.2 Simulating Copolymerization with Langevin Dynamics

We generate 7200 monomers, including free monomers and monomers in seed oligomers, in a 50σ × 50σ × 50σ periodic boundary box for all simulations. The initial monomer density is low enough that no microphase separation happens among monomers, as is appropriate for a solvated polymerization reaction. The numbers of monomer **A** and **B** are equal to each other. The system is first relaxed with only steric interaction until seeds and monomers are randomly distributed, after which attractions between type-**1** beads are introduced, and the reaction proceeds. If the distance between type-**2** beads is shorter than the bonding distance, *d*_bond_, then an intermolecular bond will form. For two type-**2** beads to get this close, they must overcome an energy barrier and be oriented in a direction favorable for reaction (see Supporting Information). The newly formed intermolecular polymer bonds act identically to those in the seed oligomers. Since the seed oligomers are reactive, free monomers have two interaction modes with the seeds: one is the reactive addition to the end of a seed, which elongates the seed oligomer, and the other is to form a non-bonded association with a seed, which is similar to the secondary nucleation in seeded supramolecular polymerization (Figure 1a).^39^ Meanwhile, it is also possible for seed oligomers to form bonds with each other. The simulation stops when the reaction extent, *p*, reaches 0.9.

Langevin Dynamics (LD) is used to simulate the dynamics of a solution environment with an implicit solvent. In LD, the total force on each monomer contains the conservative force, *F*_c_, a frictional drag term, *F*_f_, and a random force from the solvent, *F*_r_. The *damp* parameter in the frictional drag term is related to the viscosity of the solvent (see the Supporting Information). It is worth noting that we set the friction coefficient to a constant value for all atoms in this model, which neglects any changes to the effective viscosity arising from the emergent chain aggregation. Unless otherwise stated, *damp* is set to 0.1 in the simulations, which corresponds to a viscosity of 0.0910 mPa·s when the mass (*m*) and distance (σ) in LJ units are taken as 200 amu and 5 Å, respectively. This viscosity is approximately one tenth of the viscosity of water^40^ and common organic solvents such as acetone^41^ at room temperature. The temperature and volumes are held constant at 300 K and 125,000 σ^3^. All simulations were performed in LAMMPS.^42^

### 2.3 Seeds of Different Sequences Are Added Prior to Polymerization

In this work, we aim to understand the effect of seeds on the interplay of self-assembly and nematic alignment of nascent oligomers when driven by relatively weak like-monomer attractions, ε_AA,BB_ = 1.0 *k*_B_ *T* and ε_AB_ = 0 *k*_B_ *T*, and sufficient chain stiffness. Twenty seed oligomers are used for all simulations in this paper. The fraction of monomers in these seeds then differs for different seed chain lengths, ranging from 3% to 11% (see Table S4 in the Supporting Information). We tested the effect of seed oligomers with alternating, diblock, and homopolymer sequences (see Figure 1b). Alternating sequences contain alternating monomers of **A** and **B** along the chain, diblock sequences contain a block of **A** combined with a block of **B** of the same length, and homopolymer sequences contain only one kind of monomer, either **A** or **B**. We also investigated variations in seed chain length, chain flexibility, and reaction conditions to gain further insights into the seeding mechanism of the reactions and how the mechanism varies with other parameters.

## 3 Results and Discussion

### 3.1 Seed Oligomers Can Impact Block-Length Distribution and Kinetics by Influencing the Self-Assembly of Oligomers

We begin by examining the effects of seeding within our system when ε_AA,BB_ = 1.0 *k*_B_ *T*, ε_AB_ = 0 *k*_B_ *T*, and *l*_p_ = 16.5 monomer, where the non-bonded attractions between like monomers, ε_AA,BB_, can drive a microphase separation into **A**-rich and **B**-rich regions,^34^ and the chains are sufficiently stiff to nematically align within the polymerization-induced aggregates.^35^ We first explore the influence of 20 30-monomer seeds on block-length distributions and kinetics, testing the effect of adding alternating, diblock, or homopolymer seed sequences, as shown in Figure 1b.

**Seed oligomers influence copolymer aggregates, changing the block-length distribution**. Figure 2 exhibits the sample structures at the early stage and at the end of reactions in (a), (c), (e), and (g). It also includes the time evolution of the block-length distributions (b, d, f, h), culminating in the block-length distribution at reaction extent *p* = 0.9 (gray shading). The first column corresponds to the results for the control case, without preformed seeds, while the other columns show the results with 20 preformed seeds of different sequences, where the red-dashed vertical lines indicate the initial block lengths in the diblock and homopolymer seeds.

The presence of alternating seeds has no significant impact on the block-length distribution (Figure 2d), as compared to the case with no seeds (Figure 2b). In both cases, the block-length distribution peaks at 13 monomers. In contrast, the presence of diblock seeds slightly shifts the characteristic peak to 16 monomers, closely matching the initial block length of the seed oligomers to 15 monomers (Figure 2f). Finally, the presence of homopolymer seeds significantly broadens the block-length distribution to the extent that a single characteristic peak can no longer be identified (Figure 2h). Additional sequence characterizations are included in the Supporting Information and further support these findings, including the root-mean-square fluctuation (Figure S1a), the sequence autocorrelation (Figure S1b), the blockiness parameter (Figure S2), and the probability of finding like neighbors (Figure S7).

**Figure 2:**
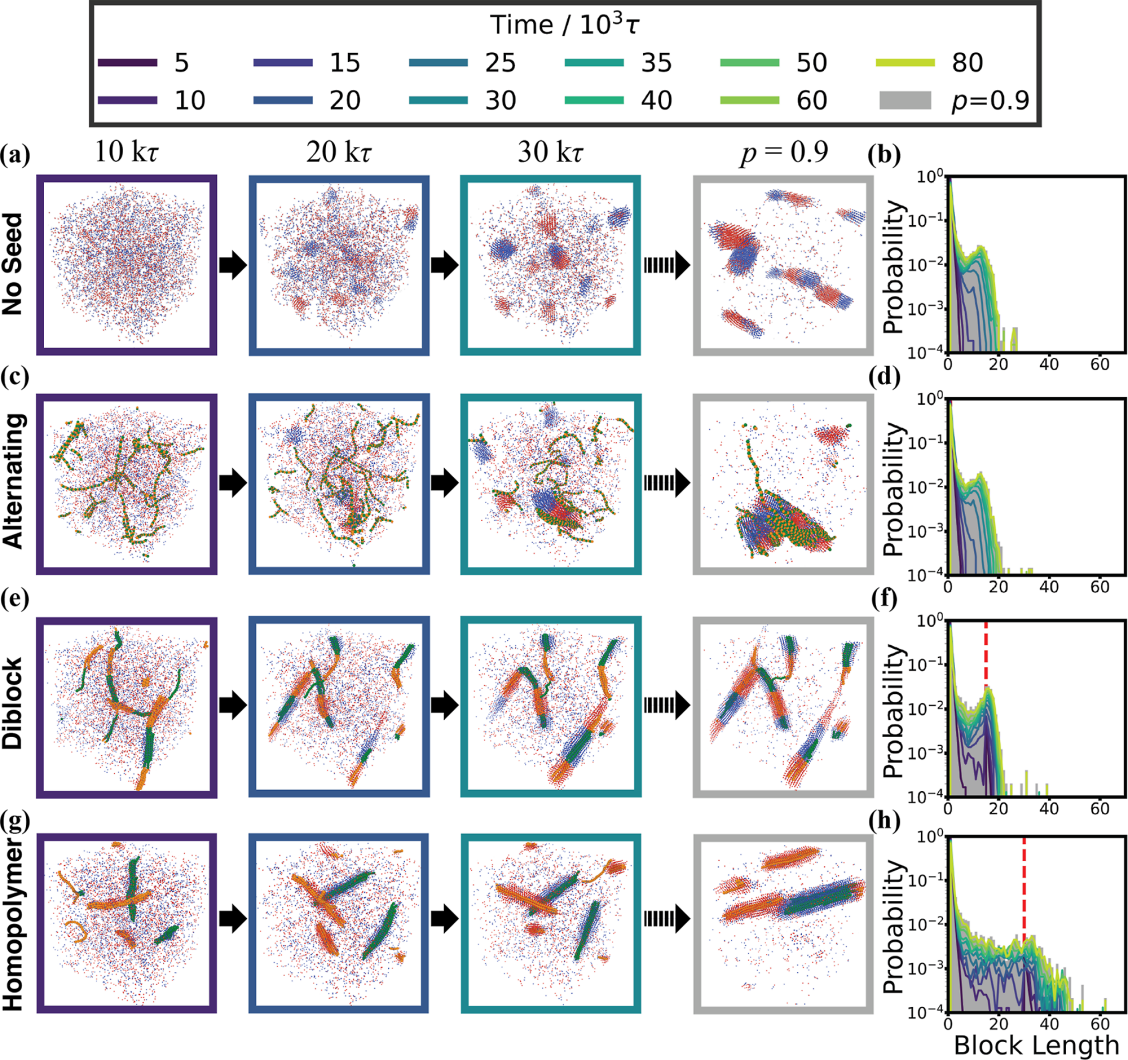
**Snapshots and block-length distributions for simulations with and without 20 seeds of length *N* = 30.** From top to bottom, the snapshots and block-length distributions are shown from simulations without seeds, with 20 alternating seeds, with 20 diblock seeds, and with 20 homopolymer seeds, respectively. From left to right, the snapshots (a,c,e,g) represent the systems at 10 × 10^3^τ, 20 × 10^3^τ, 30 × 10^3^τ, and reaction extent, *p* = 0.9. The color of the frames indicates the corresponding time of the snapshots. Red and blue beads represent the **A** and **B** monomers, respectively, while seed monomers are shown in orange (**A**) and green (**B**). For ease of visualization, the seed monomers are also enlarged in these images. The right column (b,d,f,h) shows the block-length distributions at *p* = 0.9 (gray background) and the evolution of the block-length distribution over time (colored lines). Red dashed lines highlight the block length of the initial seeds for the diblock and homopolymer seed cases.

The corresponding time evolution plots of these block-length distributions (colored lines in Figure 2d,b,f,h) as well as the snapshots near the start of the reactions (Figure 2a,c,e,g) provide insight into how the seeds influence the block-length distribution during the reactions. In the simulations without seeds (Figure 2b) and with alternating seeds (Figure 2d), a peak initially appears at approximately 10 monomers at time *t* = 25 × 10^3^τ and is further extended to 13 monomers as the polymerization proceeds. In systems with diblock seeds, a sharp peak is initially present at 15 monomers due to the block length of the preformed seeds (Figure 2f). As the reaction proceeds, this peak is moderately enhanced and broadened but only shifts slightly to 16 monomers. Furthermore, the presence of homopolymer seeds yields an initial sharp peak at 30 monomers due to the block lengths of the homopolymer seeds. However, the block-length distribution significantly broadens, and the peak rapidly disappears as the polymerization proceeds (Figure 2h).

The different block-length distributions might be attributed to the aggregates of seeds and the interaction between newly formed chains and seeds with different sequences. Diblock and homopolymer seeds form clusters at the beginning of the reactions (Figure 2e,g). The seed clusters attract monomers with the same type on their surfaces, triggering a phase separation at around 10 × 10^3^τ which is much earlier than for the simulations without seeds (Figure 2a) and with alternating seeds, where the phase separation begins around 20 × 10^3^τ. As the reaction proceeds further, the diblock and homopolymer seeds stay in the aggregate cores surrounded by newly formed chains (Figure 2e,g), guiding the chains as sequence templates to produce block-like or homogeneous clusters. This effect could explain why the addition of diblock and homo seeds has a stronger effect on the sequences of non-seeded chains than alternating seeds. Even the earliest clusters of the preformed diblock seeds display distinct **A**–**B** interfaces that template further interface growth and limit the lengths of growing **A** and **B** blocks (Figure 2e). This limit, which is introduced through the interconnected aggregates, ensures that the final peak of block-length distribution remains close to the block length of diblock seeds. However, the homopolymer seeds align with other seeds that have the same identity, either all-**A** or all-**B** chains (Figure 2g). Without any initial **A**–**B** interface, there is no inherent limit on the growth of **A** or **B** blocks, resulting in the significant broadening of the block-length distribution and the formation of longer all-**A** and all-**B** blocks than seed blocks in the simulations with homopolymer seeds.

These results demonstrate that the presence of seeds can significantly impact the self-assembly and alignment of newly formed polymer chains within the resulting clusters as a result of the sequence-dependent aggregation behaviors of the seeds, which in turn influences the final block-length distribution.

**Seeds can enhance the acceleration of reactions by increasing the nematic alignment.** To further investigate the influence of seed oligomers on the polymerization process, we have also examined the effect of seeds of these three different sequences on the reaction kinetics, as quantified by the degree of polymerization *X*_*n*_, chain dispersity *Đ*, and local nematic alignment $\overset{®}{S_{local}\;}$ vs reaction time. The results are shown in Figure 3.

**Figure 3:**
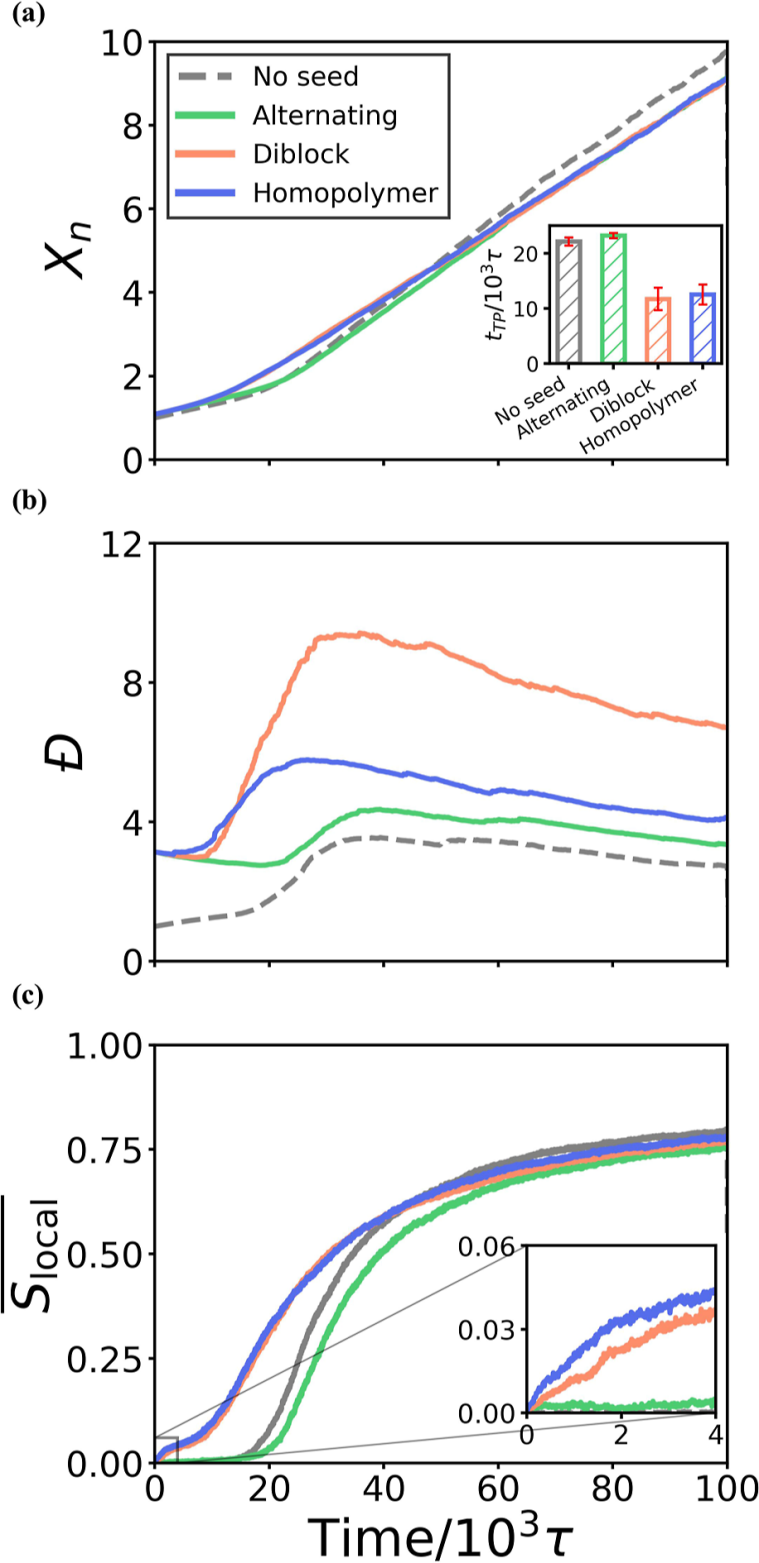
**Polymerization kinetics and nematic alignment of simulations with and without seeds.** (a) Degree of polymerization, *X*_*n*_, as a function of time. The inset figure shows the turning points, *t*_TP_, where the slope shifts for each sequence. (b) Dispersity, *Đ*, as a function of time. (c) Local nematic alignment, $\overset{®}{S_{local}\;}$ , as a function of time. In all panels, each line represents results that have been averaged across three independent simulations. Seeds are 30 monomers in length, *l*_p_ = 16.5 monomer, and the seed oligomers are included when calculating these quantities.

Under ideal conditions, where reactant concentrations remain homogeneous during step-growth polymerization, the reaction rate constant is expected to be static, and *X*_*n*_ is expected to linearly increase with time according to Flory’s principle of equal reactivity.^43^ In all cases, the growth of *X*_*n*_ was observed to be non-linear (Figure 3a), which is consistent with the previous results^34^ in this interaction regime where like-monomer attractions are strong enough to give rise to a microphase separation. The microphase separation of monomers and oligomers increases the effective rate constant and causes a breakdown of Flory’s principle of equal reactivity, as expected when the reaction mixture becomes inhomogeneous.^43^ The presence of seeds with different sequences can influence the turning point of *X*_*n*_, the time at which the change in the effective rate constant occurs (Figure 3a, inset figure). See the Supporting Information for details on the calculation of the turning point. When diblock or homopolymer seeds are present, these turning points appear at an earlier time than in the control simulation without seeds. The turning point for copolymerizations with alternating seeds is the same as that of the control. According to our previous studies, the acceleration of the reaction arises from the aggregation of growing oligomers.^34^ Such a microphase separation is also observed in polymerization-induced self-assembly (PISA).^44^ The onset of aggregation in PISA requires the block degree of polymerization (BDP) to be above a critical BDP value.^44,45^ We have found that the BDP differences between the values at *t* = 0 and at the turning point are decreased by the addition of diblock or homopolymer seeds (see Figure S6 in the Supporting Information), so that less time is needed to reach the critical threshold for the onset of aggregation and autocatalysis in these cases than in the other two cases, resulting in earlier turning points in *X*_*n*_ for diblock and homopolymer seeds.

The presence of seed oligomers can also influence the chain length dispersity, , as shown in Figure 3b. *Đ* is defined as the ratio of the weight-average molecular weight to the number-average molecular weight. In the ideal case, where Flory’s assumption holds true that reactions proceed in a chain-length independent manner, is expected to grow monotonically and plateau at a value of two.^43,46,47^ In all the cases simulated, however, we observe a non-monotonic growth of *Đ* over time, so that rapidly increases past 2, peaks, and then decreases as the reaction proceeds. The non-monotonic growth of *Đ* suggests that in the early stage, reactions between longer chains are preferential, resulting in *Đ* values that are larger than 2. However, as the reaction proceeds further, reactions between long chains and short chains become more preferential, decreasing the dispersity.^38,48^

The addition of a set of preformed seeds of exactly the same length alongside mostly monomers results in a larger initial for the seeded polymerizations. Shortly after the reaction begins, however, this value gradually drops. At the beginning of the reactions with seeds, the monomers connect to form oligomers, which decreases the overall mismatch in the chain lengths of the preformed seeds as compared to the shorter oligomers that have formed directly from monomers, reducing the overall dispersity. Once oligomers begin to locally align, the values of for the simulations with preformed seeds become larger than those without seeds. The peak and final values of vary across sequences and are the highest in the presence of diblock seeds, followed by homopolymer seeds and alternating seeds. The unusually high peaks of in the presence of diblock seeds suggest the formations of longer-than-expected chains at around *t* = 30 × 10^3^τ, which appears to arise from the merging of **A** and **B** blocks from different seed clusters. As a result, seeds aggregate into offset bundles longer than a single seed (Figure 2e), which yields especially long chains. As the polymerization proceeds further in the presence of diblock seeds, decreases but still remains relatively high compared to other cases. These results suggest that although the presence of diblock seeds can assist the control of the characteristic block length, it may generate chains that are overall less uniform in length. This conclusion is validated by the observed chain length distributions (see the Supporting Information).

In addition, Figure 3c demonstrates that the presence of diblock or homopolymer seeds induces the earlier nematic alignment of oligomers within their clusters, while alternating seeds delay nematic alignment of oligomers, compared to the control simulations without seeds. The nematic alignment of chains is measured by a localized nematic order parameter, $\overset{®}{S_{local}\;}$ , which, for this system, ranges from 0 for fully randomly oriented to 1 for fully aligned chains (see Supporting Information). With diblock or homopolymer seeds, $\overset{®}{S_{local}\;}$ increases to above 0.03 after only 1,000 τ, exhibited as shown in the inset figure of Figure 3c. These early increases in $\overset{®}{S_{local}\;}$ are attributed to the initial alignment of the diblock and homopolymer seeds (see Figure 2e,g). As the reactions proceed, these seed aggregates induce the alignment of growing chains, which increases $\overset{®}{S_{local}\;}$ further. In contrast, the alternating seeds do not align well with each other during the early stage of the reaction (Figure 2c), thus retarding the nematic alignment of nascent oligomers as compared to the case with no seeds. The differences in early stage aggregation behavior across the different seed sequences map to the way sequence variations in copolymers can affect the compatibility of copolymer chains containing both A and B monomers. The longer the all-**A** or all-**B** blocks, the stronger the driving force will be toward aggregation (see Figure 7 in ref 34), while interrupting a block of one type with a monomer of the other is expected to reduce this effective driving force.^49^ As a result, the diblock chains and homopolymers tend to generate early aggregates with other blocks of the same monomer type and increase the order parameter. While $\overset{®}{S_{local}\;}$ reaches approximately the same values at 60 × 10^3^τ in all cases, the earlier alignment of seed clusters observed for diblock and homopolymer seeds biases the synthesis of chains toward those with similar sequences and orientations.

In summary, the addition of preformed seeds with diblock or homopolymer sequences results in an earlier acceleration of the polymerization and an earlier and larger increase in *Đ*. The seeds with different sequences exhibit different degrees of alignment, which impacts when and how the newly formed chains align and assemble, thereby influencing overall sequence development during copolymerization.

### 3.2 The Effect of Seeding Depends on Seed Length, Chain Flexibility, and Block Length

We now consider how the effect of seeding varies when seed length and seed persistence length, *l*_p_, change. In this section, we examined simulations with seed lengths ranging from 10 to 40 monomers and *l*_p_ values ranging from 6.0 to 16.5 monomers.

**The effect of seeding depends on chain length.** We first tested how the effect of adding seeds varies with the seed chain length. Figure 4 compares the block-length distributions resulting from the addition of seeds of different sequences and seed lengths to that from the control simulations with no seeding (gray shading). Varying seed length clearly influences the resulting block-length distribution in a sequence-dependent manner. For seeds with an alternating sequence, the block-length distribution is not sensitive to changes in seed lengths (Figure 4a); however, their effect on *p*_AA,BB_ is dependent on seed length (see Figure S7).

**Figure 4:**
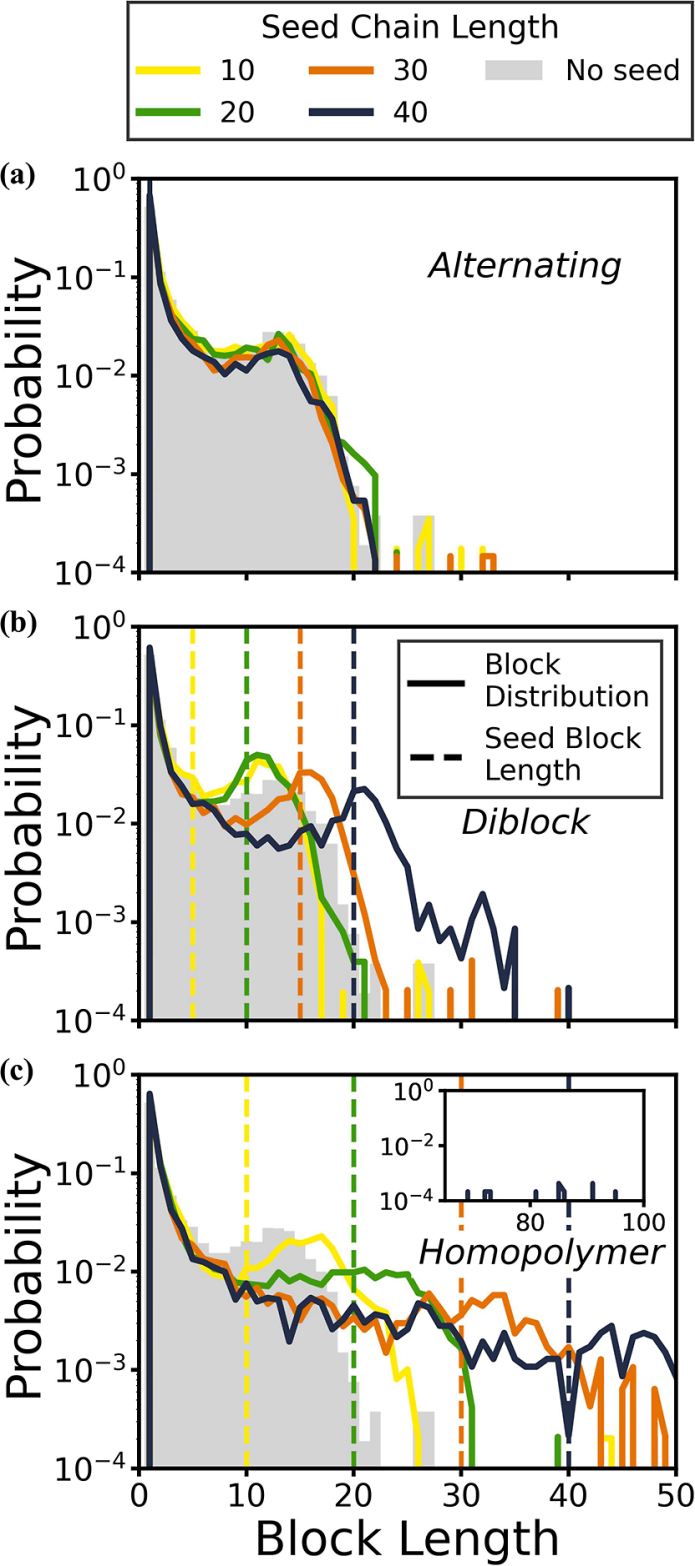
**Block-length distributions from polymerizations with seeds of differing sequences and seed lengths.** Results are shown for simulations with (a) alternating seeds, (b) diblock seeds, or (c) homopolymer seeds. Seed lengths were varied from 10 to 40 monomers, and the seeded results are plotted over those for the unseeded case (gray shading). The dashed lines in the figures indicate the initial block lengths within the seeds (not shown for alternating sequences). Results are averaged over three independent trials, and *l*_p_ = 16.5 monomers.

In the cases of diblock seeds, when the seed chain length is above 20 monomers (with a block length of at least 10 monomers), increasing the seed length increases the characteristic block length to a value close to the block length in the initial seeds (Figure 4b). In the cases of homopolymer seeds, the block-length distribution is significantly broader than that in the control simulations. Although there is a characteristic peak around 16 monomers when the length of the homopolymer seeds is equal to 10 monomers (Figure 4c, yellow line), the final block-length distribution with the longer homopolymer seeds shifts to the right and broadens, no longer exhibiting an identifiable peak (see in Supporting Information). The preference toward longer blocks, as the diblock or homopolymer seeds lengthen, is also reflected in the increase of *p*_AA,BB_ in the Supporting Information, Figure S7.

**The effect of diblock seeds depends on chain flexibility.** In Figure 4, we observe that as the chain length of the diblock seeds lengthens past 20 monomers (with a corresponding block length of 10 monomers or more), the characteristic peak of the resulting block-length distribution increases to match the seed block length. To probe how this correlation depends on chain flexibility, we ran the same copolymerizations in the presence of 20 diblock seeds with varying chain lengths (*N* = 10, 16, 20, 30, and 40 monomers) and varying persistence lengths (*l*_p_ = 6.0, 9.9, and 16.5 monomers). The results shown in Figure 5 demonstrate that the block-length distribution is sensitive to both the seed chain length and *l*_p_.

**Figure 5:**
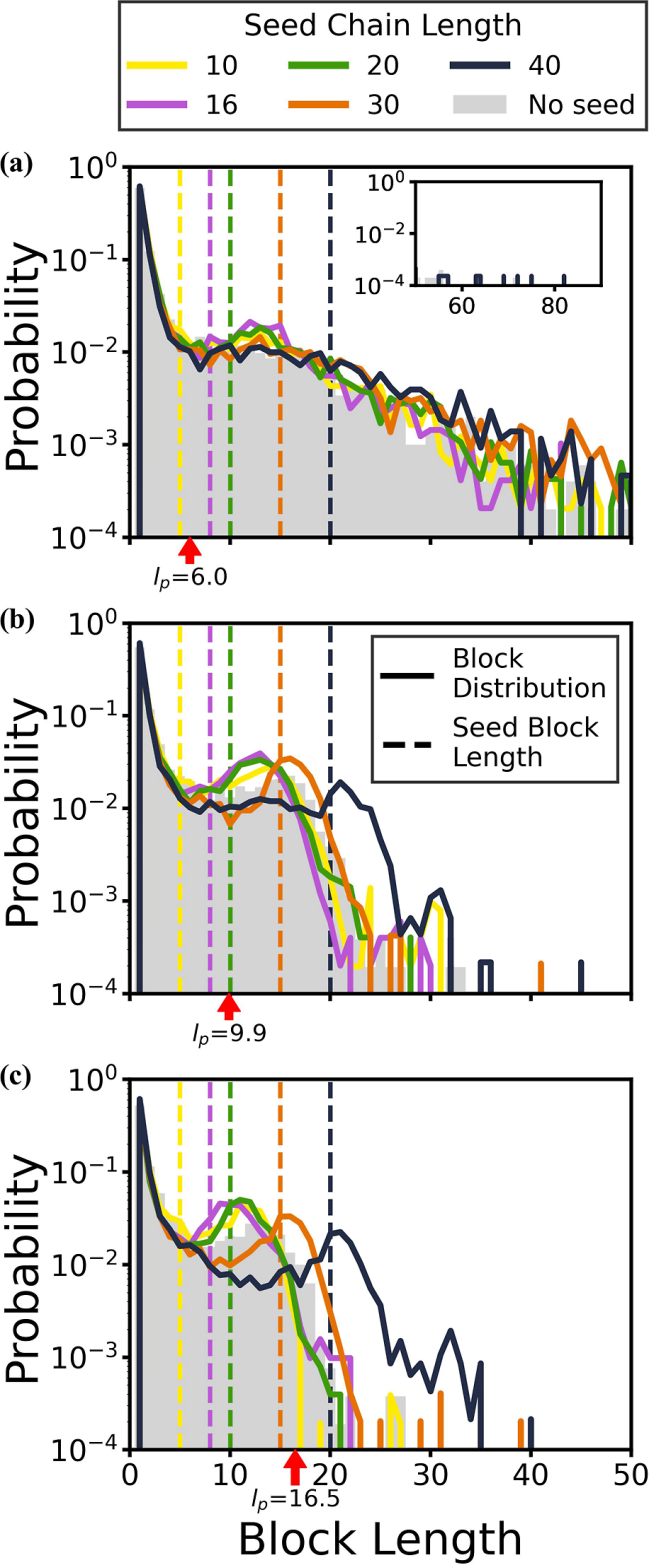
**Block-length distributions of oligomers with different persistence lengths, *l*_p_, from simulations with diblock seeds of varying lengths.**The red arrows on the *x*-axes indicate the *l*_p_ for that set of copolymers, which is 6.0 monomers in (a), 9.9 monomers in (b), and 16.5 monomers in (c). The dashed lines in the figure indicate the initial block length of the diblock seeds, which is a half of the seed length shown in the legend. Results from simulations with the appropriate *l*_p_ without seeds are shown in the gray shading.

In the case of no seeds, as we increased *l*_p_ from panels (a) to (c), the block-length distribution narrows, and the peaks become more defined. The appearance of a characteristic non-zero peak when ε_AA,BB_ = 1 *k*_B_ *T* and ε_AB_ = 0 for sufficiently stiff chains is consistent with earlier results on this model^35^ and arises from the interplay of the microphase separation and the nematic alignment of nascent oligomer chains.^35,38^ We term this process kinetic self-templating growth,^38^ as the nematically aligned aggregates serve as templates for further growth.^38^

As the stiffness of the copolymers increases, this self-templating mechanism is enhanced, thereby affecting the resulting block-length distributions. When the chains have a reduced stiffness (*l*_p_ of 6.0 monomers in Figure 5a), there is no obvious characteristic block length, and the distribution is most spread out for longer seeds. As the *l*_p_ increases (to 9.9 monomers in Figure 5b), characteristic block lengths emerge and an alignment between the resulting characteristic block lengths and the initial seed block lengths appears for longer seeds. We can define a threshold seed block length for a given *l*_p_ as the seed block length, where the final characteristic block length is within 20% of the initial seed block length. Using this descriptor, the threshold seed block length at *l*_p_ = 9.9 monomers is between 10 and 15 monomers. At the larger *l*_p_ value of 16.5 (in Figure 5c), the threshold seed length shifts to a value between 5 and 8 monomers (see in Supporting Information). The correspondence between the initial seed block length and the final characteristic block length continues to improve as the chain becomes stiffer for the seeds over this threshold seed block length value. Together, these results demonstrate that the presence of diblock seeds with sufficient lengths can increase the formation of chains with blocks that match the initial seed block lengths and that the seeding effects of diblock seeds can be enhanced by increasing the copolymer chain stiffness.

The improved seeding effects of longer stiff seed oligomers can be attributed to the early aggregation and alignment of seeds, as exhibited in the time traces of the local nematic order parameter in the systems with varying seed lengths and stiffnesses (see Supporting Information, Figure S11). Since longer chains have more monomers aligned in the nematically ordered aggregates than the shorter chains, their alignment is more enthalpically favorable. At the same time, for a fixed number of monomers, the entropic cost of alignment is less in a set of longer chains than in a set of shorter chains since the longer chains have already tied more monomers together. It is important to note that this seeding effect only appears once the *l*_p_ reaches a certain value. We find that for *l*_p_ values of around 6.0, longer chains are simply too flexible to nematically align and regulate the block lengths of the forming chains. We further tested diblock seed oligomers with different persistence lengths for the **A** blocks and **B** blocks, and the results show that seed oligomers could be employed to regulate the length of both stiff and flexible blocks, demonstrating the potential application of this method for rod–coil polymers (see Supporting Information Figure S13).

**The effect of seeding depends on the block lengths within those seeds.**In order to investigate the seeding effect across a range of sequence length scales,^50^ in Figure 6, we vary the seed block length from 1 to 20 monomers within seeds with a constant chain length of 20 monomers.

**Figure 6:**
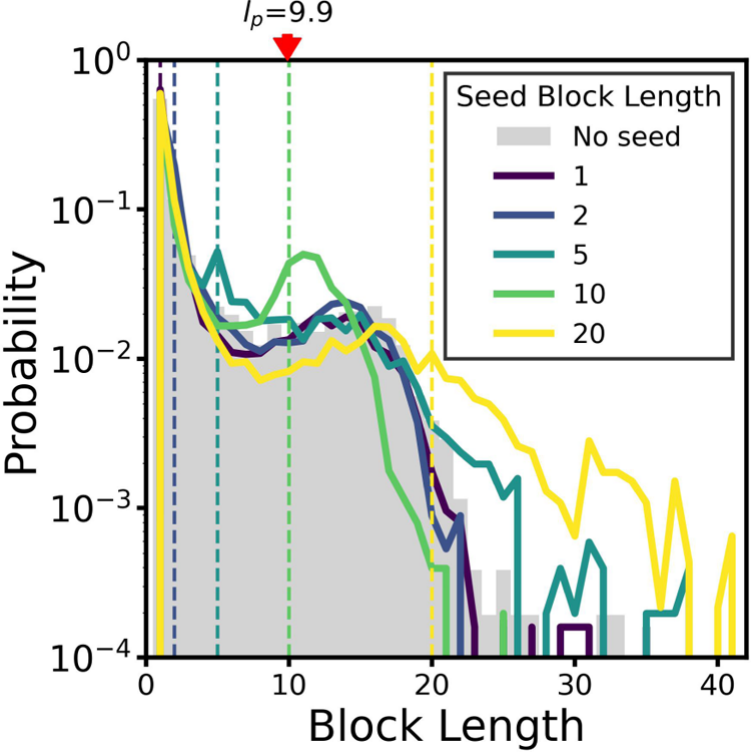
**Block-length distributions from simulations with varying seed block lengths.**The seed oligomers in these simulations all have the same chain length at 20 monomers, while the block length within each seed varies from 1 to 20 monomers, as indicated by the dashed vertical lines. Twenty seeds with a persistence length of 9.9 monomers are used in each simulation, and results are shown from three independent trials.

The final block-length distribution is not significantly influenced by seeds with very short block lengths (1–2 monomers), which is far shorter than both the persistence length of 9.9 monomers and the block-length distribution peak in the case with no seeds (15 monomers). As the seed block length increases to 5 monomers, the characteristic block length of newly formed chains deviates from the result without seeds and aligns with the seed block length, but the distribution still shows a strong shoulder at around the no-seed peak of 15 monomers. Interestingly, at seed block lengths of 10 monomers, the distribution peak shifts to 11 monomers, just longer than the seed block length. At seed block lengths of 20 monomers, however, the distribution peak shifts to the right with a maximum value slightly above the no-seed distribution peak. In this case, the block-length distribution is also broader than those of all other simulation results.

There are many factors that contribute to these results. Seeds that are long enough to enable good alignment among the different blocks when they aggregate have a favorable enthalpic driving force for aggregation, providing more effective templates. Block lengths that are long enough to enable the formation of stable interfaces within these templates (such as those found in the simulations with seed block lengths of 10 monomers; see Figure S14) yield final block-length distributions with defined peaks that lie close to the seed block length but shifted toward the peak of the no-seed case. In addition, the number of interfaces in the seeds may also play an important role. Seeds with blocks of 20 monomers have no interfaces since the seed length itself is 20 monomers and thus yield a much broader distribution of final block lengths. Moreover, seed blocks that are longer than the persistence length can form structures that fold, reducing alignment within the aggregate and possibly weakening the templating effect (see Figure S14e in the Supporting Information).

### 3.3 The Effect of Seeding Mitigates the Effect of Diffusion and Reaction Time Scales on Block-Length Distributions

Having established the effect of seed chain length and stiffness on the resulting sequences, in this section, we explore the effect of solvent viscosity and activation energy on block-length distributions. To do so, we vary the solvent viscosity from η = 0.0091 to 0.91 mPa·s and compare the final block-length distributions from simulations with and without seeds in Figure 7. We then vary the activation energy from 6.8 to 9.8 *k*_B_ *T* and compare the block-length distributions in Figure 8.

**Figure 7:**
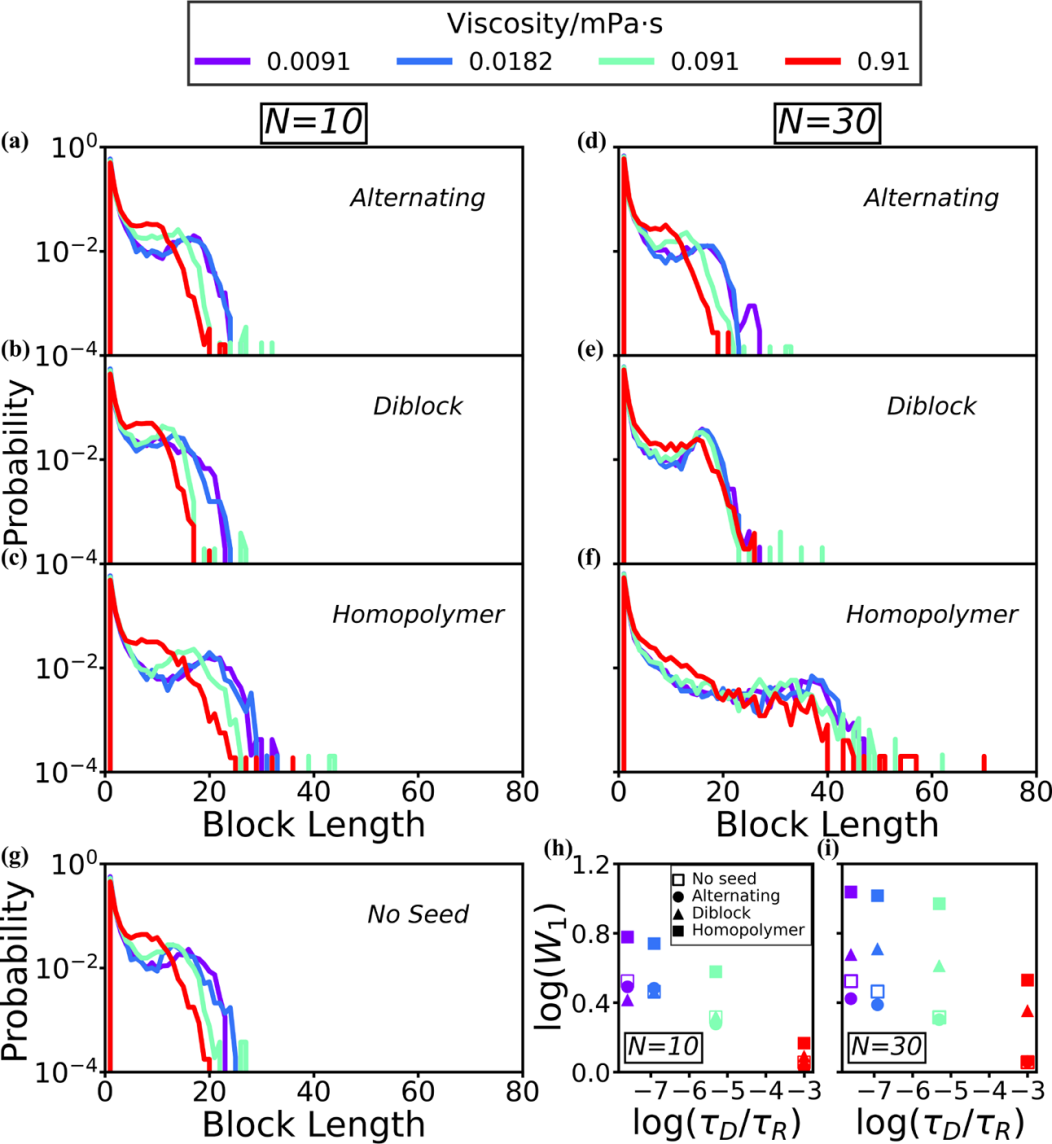
**Block-length distributions from simulations with varying viscosity for different seed sequences and lengths.** (a–f) Block-length distributions are shown for copolymerizations with alternating seeds (a,d), diblock seeds (b,e), and homopolymer seeds (c,f), across a range of viscosities. The results are shown for both seed lengths of 10 monomers (a–c) and 30 monomers (d–f). In (g), we show the block-length distribution for these same viscosities without seeds. (h,i) The Wasserstein distance, (*W*_1_), was calculated between the block-length distributions obtained from simulations and those predicted from Markovian statistics (see Supporting Information). Here, we plot log­(*W*_1_) vs log­(τ_D_/τ_R_), where (τ_D_/τ_R_) is the ratio between the diffusion and reaction time scales^38^ for seed lengths of 10 (h) and 30 (i).

**Figure 8:**
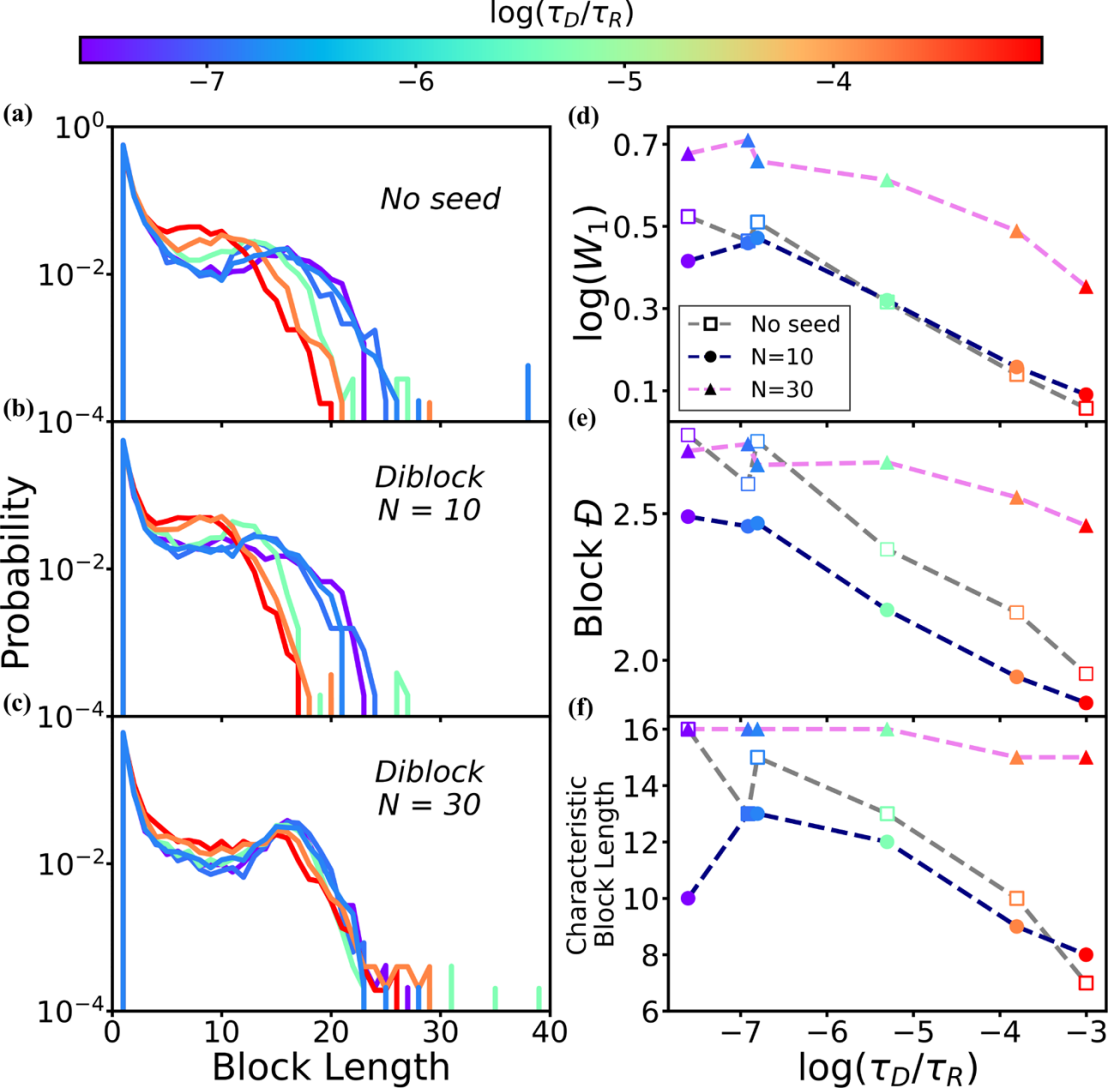
**Block-length distribution for simulations with different ratios of diffusion and reaction time scales, performed with diblock seeds of varying lengths.** (a–c) Block-length distributions are shown both without seeds (a) and with 20 diblock seeds of 10 monomers (b) and 30 monomers (c). The lines in (a–c) are colored according to the time scale ratio, specifically log­(τ_D_/τ_R_). We also plot (log­(*W*_1_)) vs log­(τ_D_/τ_R_) in (d), block dispersity (block *Đ*) vs log­(τ_D_/τ_R_) in (e), and the characteristic block length vs log­(τ_D_/τ_R_) in (f) for simulations without seeds (squares, gray dashed lines), with 10-monomer diblock seeds (dots, navy dashed lines), and with 30-monomer diblock seeds (triangle, violet dashed lines). The Wasserstein distances are calculated by comparing the real block-length distributions with those predicted from Markovian statistics (see Supporting Information).

In a previous study,^38^ we found that the characteristic block length shifts toward a larger value as the solvent viscosity decreases and the activation energy increases for the simulations without seeds. The effect from solvent viscosity and activation energy is considered collectively by combining the effective reaction and diffusion time scales, τ_D_/τ_R_ (see Supporting Information). As τ_D_/τ_R_ decreases, the block-length distribution shifts further from a Markovian distribution, showcasing the linked influence on the final sequence biasing of the activation barrier and reactant diffusivity.

**Seed oligomers and viscosity regulate the block-length distributions.** We start our analysis by comparing the simulation results across varying viscosities for all seed sequences. To quantify the effect of viscosity, we utilized the Wasserstein metric, *W*_1_, between the final block-length distribution and the ideal distribution using Markovian statistics for the given *p*_AA,BB_ observed in that specific simulation (further described in ref 38). The more the simulated block-length distribution deviates from the ideal Markovian distribution, the larger the value of the Wasserstein distance that we obtain.

For the simulation without seeds (Figure 7g), the results are consistent with our earlier work,^38^ where log*W*_1_ decreases linearly with log­(τ_D_/τ_R_) (open squares in Figure 7h,i). In this set of simulations, we therefore see that the characteristic block length shifts toward shorter blocks as the viscosity increases (Figure 7g).

For systems that initially contain short, 10-monomer seeds at viscosities above 0.0182 mPa·s, the characteristic block length also shifts to the left for all seed sequences as viscosity increases (Figure 7a–c). Therefore, the Wasserstein distance decreases to a similar extent as in the case without seeds (Figure 7h, filled shapes). However, when the viscosity is below 0.0182 mPa·s, the block distributions are not significantly influenced by the change of viscosity, as indicated by the similar Wasserstein distance values when the viscosity is 0.0091 and 0.0182 mPa·s (Figure 7h, purple and blue).

Interestingly, once the seed chain length is increased to 30 monomers, the sequence of the seed influences how it responds to changes in viscosity. The block-length distributions (Figure 7d) and log*W*_1_ (Figure 7i, dots) for the simulation with alternating seeds are almost exactly the same as the results with shorter alternating seeds (Figure 7a). However, for longer, 30-monomer diblock seeds, the characteristic block lengths remain almost unchanged across all viscosities (Figure 7e). Correspondingly, the log*W*_1_ obtained from the simulation with longer diblock seeds has no significant change until the viscosity is over 0.091 mPa·s (Figure 7i, triangles). As for the longer, 30-monomer homopolymer seeds, the resulting block-length distribution broadens to the extent that it is difficult to identify a characteristic length. Therefore, the value of log*W*_1_ is the largest among all simulations (Figure 7i, filled squares). Even though there is no obvious characteristic block length, the block-length distributions in Figure 7f overlap when the viscosity is below 0.091 mPa·s (purple, blue, and green lines). In general, for all 30-monomer seed sequences, the probability of forming shorter blocks increases significantly at the higher viscosity of 0.91 mPa·s, while the probability of longer blocks decreases, such that there is a significant drop in log*W*_1_ at this viscosity (red filled shapes in Figure 7i).

More constant block-length distributions and steadier values of log*W*_1_ are observed for the longer diblock and homopolymer seeds in Figure 7i. When the viscosity is low, the seeds and the growing oligomers have enough time to align and form energetically favorable nuclei with distinct domains before the next polymer bond forms. Therefore, the templating effect arises from both self-templating and seeding increases at lower viscosities, as can be seen with the consistent shift from left to right with the red to purple. For longer diblock and homopolymer seeds, the templating effect is less influenced by changes in viscosity, possibly due to the increased contact surface area of each domain, which makes it easier for small oligomers and monomers to find and aggregate around the seeds.

**Longer diblock seeds dampen the effect of viscosity and reaction times on the final sequence.** Given the interesting differences in block-length distributions for diblock seeds of varying lengths, as seen in the comparison of Figure 7b,e, we further investigate the effect of these diblock seeds to study the interaction between relative kinetic time scale, τ_D_/τ_R_, and seed length by varying both the solvent viscosity and activation barrier. The results are shown in Figure 8, where we plot the block-length distributions in panels (a–c). To capture the broadening of distributions, we calculate the log­(*W*_1_), as before in panel (d), as well as the block dispersity, which is represented as block *Đ* in panel (e) and the characteristic block lengths in panel (f).

As mentioned before, without seeds, the block-length distribution shifts to the left when τ_D_/τ_R_ increases (Figure 8a), resulting in an overall decrease in log*W*_1_, block *Đ*, and characteristic block length (Figure 8d–f, gray lines with unfilled squares). The addition of diblock seeds with a length of 10 monomers leads to narrower block-length distributions (Figure 8b) compared to the results without seeds, which is further supported by lower block *Đ*s and shorter characteristic block lengths (navy lines with filled dots in Figure 8e,f). However, the log*W*_1_ from simulations with 10-monomer diblock seeds is generally similar to those obtained without seeds. In contrast, the addition of diblock seeds with a longer length of 30 monomers results in a minimal shift in the block-length distribution (Figure 8c), with a characteristic block length of 15–16 monomers in all cases (Figure 8f, pink line with triangles). The log*W*_1_ and block *Đ* values decrease more gradually as compared with the shorter seeds (Figure 8d,e, pink line with triangles). The elevated log*W*_1_ values indicate that these longer diblock seeds enhance the sequence biasing overall. Taken together, these observations support the conclusion that the seeding effect of longer, 30-monomer diblock seeds is more robust and less affected by the change of τ_D_/τ_R_.

## 4 Conclusion

In this article, we have studied the effect of adding seed oligomers of varying sequences to the reaction mixture prior to the start of polymerization, with approximately 3–11% of the monomers included in the seeds. We examined the impact of these seeds on the reaction kinetics and the resulting block-length distributions. When the non-bonded interaction between like monomers is strong enough, the presence of diblock and homopolymer seed oligomers significantly influences the final block-length distribution. However, the effect of alternating seeds is minimal such that polymerizations with alternating seeds yield almost identical results to those without seeds. The autoacceleration of the reaction occurs earlier in the presence of diblock and homopolymer seeds, which is a result of the earlier phase separation of monomers and alignment of oligomers in these systems. We also find that the effect of the preformed seeds depends on seed chain length, block length within the seed, and chain flexibility. Although the block-length distribution is not influenced by the lengthening of alternating seeds, the final block-length distribution shifts to the right as the seed chain lengths increase for diblock and homopolymer seeds. In the simulations with stiffer diblock seeds over about 20 monomers in length, the characteristic block length mirrors that of the initial seed block length, even over a wide range of relative kinetic time scales. The characteristic block length can also align with the seed block lengths when they are in a certain range around the persistence length. Finally, the presence of seeds greatly reduces the changes in block-length distribution that otherwise occur with changes in viscosity, especially when the viscosity is low and the seed chain length is long.

The results from this work provide a new and potentially simple route to influence the sequence of copolymers via the addition of preformed, sequence-defined oligomers in step-growth copolymerizations. The influence of these seed oligomers on sequences is evident even when the fraction of seed monomers is as low as 2.7%. Depending on the specific polymers under consideration, it may be possible to select and tune parameters such as chain flexibility, solvent selectivity, solvent viscosity, and seed length and sequence to achieve more control over statistical copolymer sequences with the help of seed oligomers. Interestingly, our results also demonstrate the potential to design preformed seed oligomers that can serve as a counterbalance to the way that changes in solvent viscosity and activation energy influence the final block-length distribution.

This work extends the existing set of interactions used for templated polymerization from oriented and strong interaction, such as electrostatic^51–53^ and hydrogen bonding,^24,52,54^ to weaker, isotropic interaction. Seeds could therefore be employed to bias copolymer products toward certain target sequences, provided those sequences have persistence lengths that promote chain alignment, as is the case for liquid-crystal copolyester^55^ and conjugating polymers.^56^ For example, diblock seeds could be used to influence the block length of synthetic diblock copolymers in one-pot reactions, providing a route to better control the micellization for applications in drug delivery,^57^ lubricants,^58,59^ and nanotechnology.^60,61^

The alignment between characteristic block lengths and seed block lengths plus the overall reaction acceleration in the presence of seeds demonstrates that seed-aided templated polymerization could provide an effective and efficient way to reduce the dispersity of blocks within copolymers. These observations could also provide interesting insight into biological polymers with blocky sequences,^62^ such as mussel foot protein,^63–65^ silks,^66^ tropoelastin,^67^ and suckerins.^68^ A seed-aided strategy for sequence biasing could help with the synthesis and engineering of blocky biomimetic materials. The new perspective provided by this framework may also help us better understand the interplay of forces that enable sequence replication in more complex and dynamic environments, such as during biological polymer sequence replication for nucleic acids^69^ and proteins.^70^

This work merely begins to probe the effect of adding preformed seeds to step-growth copolymerization. Future work is needed to investigate the interplay between these and other factors that may influence the seeded templating effects observed here for more complicated sequence patterns and within specific copolymer reactions.

## Supplementary Material



## Notes

Supplementary materials include model details, further characterization of copolymer sequences, data analysis processes for the identification of turning points and characteristic block length, parameters from the simulations with seed oligomers, and simulation results with seed oligomers of asymmetric persistence lengths (PDF)
