# Supplementary material for: Seed Oligomers Regulate Sequence Development through a Templating Effect in Simulated Irreversible Step-Growth Copolymerization
Source: Macromolecules. 2025 Dec 5;58(24):13478–90. doi: 10.1021/acs.macromol.5c01639 (PMC12752685; doi:10.1021/acs.macromol.5c01639)
Supplement: Supplementary file 1 [file ma5c01639_si_001.pdf]

Supporting Information for *Seed Oligomers Regulate Sequence Development through a Templating Effect in Simulated Irreversible Step-growth Copolymerization*

Wenxin Xu[†,§] Nhu Q. Nguyen[†,‡,§] Kateri H. DuBay[*,†]

† Department of Chemistry, University of Virginia, Charlottesville, Virginia 22904, United States
‡ Fulbright University Vietnam, Ho Chi Minh City, Vietnam
§ These authors contributed equally to this work.

S1 Methods

The model used in this paper was previously developed by the DuBay group[1,2] built with LAMMPS.[3] We include below the detailed description of the simulation settings.

S1.1 Interaction

Non-bonded interactions The non-bonded interactions between center particles are governed by a modified Lennard-Jones potential as stated in the main article.

As for the linker (type-**2**) particles, a soft short-ranged repulsion is constructed:

$$E_{(2,2')} = \begin{cases} \frac{h}{2}cos(\frac{\pi(r-d_{\text{bond}})}{d_{\text{onset}}-d_{\text{bond}}}) + \frac{h}{2} & d_{\text{bond}} < r < d_{\text{onset}} \\ 0 & r \geq d_{\text{onset}} \end{cases} \tag{S1}$$

The d_{bond} and d_{onset} refer to the bonding and interaction onset distances which are 0.2 σ and 0.3 σ respectively. With the repulsive interaction between type-**2** particles, the activation energy is adjustable via tuning the h parameter. The h parameters and the correlated observed activation energy values are listed in Table S1. The observed activation energy values are measured in bonding events in our previous work with sampled simulations.[2,4]

Table S1: Simulation observed activation energy in the unit of $k_{\text{B}}T$ for different h parameter in Equation S1.

h	**Activation Energy** / $k_{\text{B}}T$
1.5	6.8
3.0	8.3
4.5	9.8

Bond potential All bonds are modeled as harmonic bonds, and described by the equation :

$$E_{bond}(r_{ij}) = K_{ij}^{bond}(r_{ij} - r_0)^2 \tag{S2}$$

where the K_{ij}^{bond} is the bond constant, and the r_0 represents the equilibrium bond length between atoms i and j. For intermolecular bonds between type-**2** particles, the K_{ij}^{bond} is 6000 $\epsilon \cdot \sigma^{-2}$, r_0 is 0.15 σ. The intramolecular bonds between type-**1** and type-**2** particles are with a K_{ij}^{bond} of 2000 $\epsilon \cdot \sigma^{-2}$ and a r_0 of 0.4 σ. Here, ϵ is the energy unit in LJ reduced units, set as 100 $K \cdot k_{\text{B}}$.

Angular potential Intramonomer and intermonomer angles are governed via the harmonic potential:

$$E_{\text{angle}}(\theta_{ijk}) = K_{ijk}^{\text{angle}}(\theta_{ijk} - \theta_0)^2 \tag{S3}$$

in which θ_{ijk} is the angle between particles i, j and k, θ_0 is the equilibrium angle and, K_{ijk}^{angle} is the spring constant for the angle. θ_0 is 180° for all angles. For the intermonomer angles, **1-2-2′**, $K_{212'}^{\text{angle}} = 100\ \epsilon \cdot \text{rad}^{-1}$ for all chains. The spring constant of the intramonomer angles between particle **2-1-2′** is the parameter relevant to the stiffness of the chains.[2] The corresponding angular constants and persistent length used in this paper is listed in the Table S2, which is from the Ref. [2].

Table S2: Persistence lengths of chains with different angular spring constant of intramonomer angles, K_{ijk}^{angle}.[2]

K_{ijk}^{angle} / $\epsilon \cdot rad^{-2}$	**Persistence length** (l_p)
10	6.0
20	9.9
50	16.5

S1.2 Dynamics

Langevin dynamics We applied Langevin dynamics to simulate a solution background. The total force on a particle consists of three parts. In addition to a conservative force from the interaction between particles, there is also a random force and a fractional drag term. The *damp* parameter in the fractional drag term is related to the viscosity of the solution:

$$\eta = \frac{m}{3\pi d \cdot damp} \tag{S4}$$

The m and the d are the mass and size of particles. For a monomer with a diameter of 5 Å and with a mass of 200 amu, the *damp* values and correlated viscosity used in this paper are summarized in the Table S3.

Table S3: Viscosities that correspond to the simulated *damp* parameters used in the Langevin dynamics implementation.

damp (τ)	**Viscosity** (η) / mPa · s
1.00	0.0091
0.50	0.0182
0.10	0.0910
0.01	0.9100

S1.3 Seed oligomers

Fraction of monomers in seeds The fraction of the monomers that are initially included as seeds depends on the seed length and are listed in Table S4. The fraction ranges from 2.78% to 11.11%, providing enough seeds to produce an effect on the final chain sequences, while also avoiding excessive monomer consumption.

Table S4: The fraction of the total 7200 monomers that are included in seed chains for different seed lengths, given the fixed number of 20 seeds in all simulations in this work.

Seed Length	Seed Number	Seed monomer fraction
10	20	2.78%
20	20	5.56%
30	20	8.33%
40	20	11.11%

S2 Seed oligomers can impact block-length distributions and kinetics by influencing the self-assembly of oligomers.

Additional parameters to characterize sequences. In order to characterize the sequence on a length scale close to the seeded block lengths, we calculate the root mean square fluctuation, $F(l)$, in Figure S1a, and the sequence autocorrelation function, $C(l)$, in Figure S1b for simulations without seeds and with twenty 30-monomer seeds.[5] We exclude all chains containing seeds from this analysis to better analyze their templating effect on nearby sequences.

For both calculations, the sequences are represented as a 1D walk, in which the **A** and **B** monomers are mapped as +1 and -1 step values ($u(i) = +1$ or -1), respectively. The 'net displacement', $y(l)$, is the summation of step values over l monomers, that is $y(l) = \Sigma_{i=0}^{l} u(i)$.

The root mean square fluctuation is then based on the difference in net displacement $\Delta y(l)$, which is defined as $\Delta y(l) = y(i + l) - y(i)$, such that:

$$F(l) = (\langle [\Delta y(l) - \langle \Delta y(l) \rangle]^2 \rangle)^{1/2}. \tag{S5}$$

For uncorrelated random sequences, $F(l)$ increases with the square root of the length in monomers, $F(l) \sim l^{1/2}$ (red dashed line in Figure S1a). However, $F(l)$ from our simulations deviates from the trend expected for random sequences, with a slope greater than 1/2 indicating long-range correlations in the final chain sequences. The simulations with homopolymer seeds deviate the most from the random sequence slope of 1/2, showing that the sequences from those simulations have the longest-range correlation.

The autocorrelation function $C(l)$, which is the expectation value of the product of step i and $i+l$:

$$C(l) = \langle u(i)u(i+l) \rangle. \tag{S6}$$

The autocorrelation functions for the simulations have a periodically changing pattern. The first minimum is related to characteristic block length, which is the most favorable block length in the final sequences. Compared with the simulation without seeds, the addition of diblock and homopolymer seeds shifts the location of the first minimum in $C(l)$ to longer lengths of 17 and 57 monomers, respectively. In contrast, the first minimum $C(l)$ for the simulations with alternating seeds is smaller than the one from simulations without seeds. These results agree with the block length distributions and the lengths of the seed blocks.

We also calculate the blockiness parameter, Λ[6,7] from the correlation parameter, λ:

$$\Lambda = \frac{1+\lambda}{1-\lambda}, \tag{S7}$$

where λ is related with the ratio of pair fractions, f_{ii}, which is equal to the ii pair count divided by all pair counts, such that

$$\lambda = \frac{f_{\mathrm{AA}}}{f_{\mathrm{AA}} + f_{\mathrm{AB}}} + \frac{f_{\mathrm{BB}}}{f_{\mathrm{BB}} + f_{\mathrm{BA}}} - 1. \tag{S8}$$

$\Lambda = 0$ for alternating sequences, while $\Lambda = 1$ for random sequences. When the Λ is significantly above 1, it represents highly blocky sequences.

The resulted Λ for pairs (excluding those in the initial seeds) are shown in Figure S2. All of the Λ values are above 1, indicating a preference to form blocky chains in all conditions. The addition of alternating seeds do not generate significant difference compared with the simulation without seeds. The addition of diblock and homopolymer seeds not only result in a higher Λ, but also accelerate the increase of Λ earlier on in the simulation. These results agree with the longer characteristic block length and broader distribution from the analysis of block lengths for these seeds. The plots also echo the earlier increase in X_n with the addition of diblock and homopolymers in Figure 3.

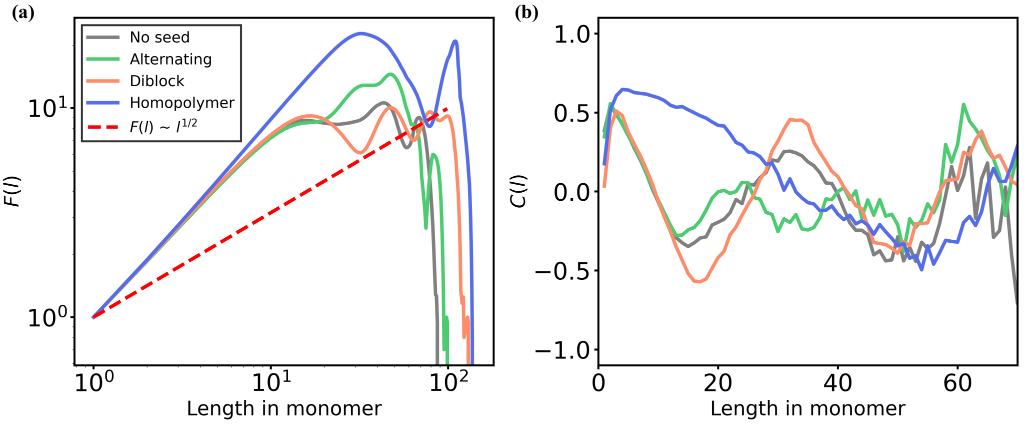

Figure S1: (a)Root mean square fluctuation and (b)autocorrelation function as a function of length in a unit of monomers for the simulations without seeds and with 20 30-mer seeds. The persistence length for chains is 16.5 monomers. The chains with seeds are excluded from calculation. The red dashed line in (a) represents the correlated random sequence with a relation as $F(l) \sim l^{1/2}$.

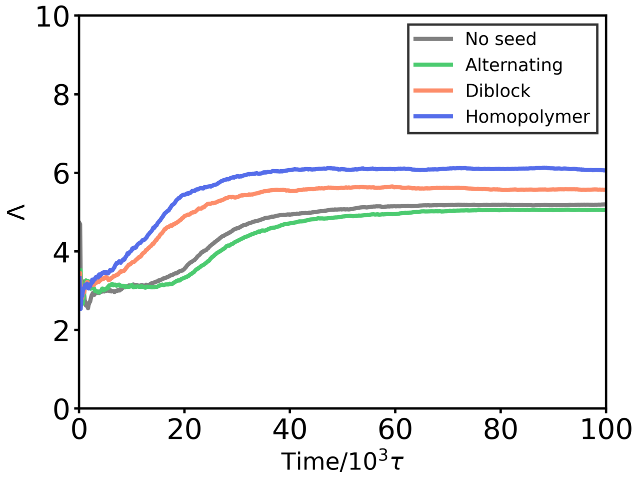

Figure S2: Blockiness parameter, Λ, evolution with reaction time for the simulations without seeds and with 20 30-mer seeds. The pairs in the seeds are excluded. The persistence length for chains is 16.5 monomers.

Order parameter calculation The local order parameter, $\overline{S_{\text{local}}}$, is a measure of how well the particles align in the same direction, calculated from the formula:

$$\overline{S_{\text{local}}} = \left\langle \frac{3\cos^2\theta - 1}{2} \cdot \mathbf{1}_{\text{aggregate}} \right\rangle \tag{S9}$$

The θ is the angle between the monomer and its local director which is defined as the average orientation of all monomers within 2.5 σ of the target monomer. The $\mathbf{1}_{\text{aggregate}}$ takes on the value of 1 for aggregated monomers with more than 12 neighbour monomers within 2.5 σ, and 0 for other unaggregated monomers.

Turning point The determination of the turning point in the plot of X_n-t (Figure 3 in the main text) is shown in Figure S3. The turning point is identified by locating the maximum in the second derivative of X_{n} before 45k τ, which is calculated from the smoothed first derivative from the Savitzky-Golay filter[8] with a window length of 15.5k τ and an order of 5. The second derivative is smoothed by Gaussian fitting with a sigma of 1.2k τ. Comparing the turning points from simulations with different seed sequences and lengths (Figure S4), we observe that increasing the diblock and homopolymer seed lengths results in an earlier turning point. The addition of alternating seeds, however, did not influence the turning points, with no change observed between the turning points in simulations with alternating seeds and in those with no seeds, regardless of the seed length.

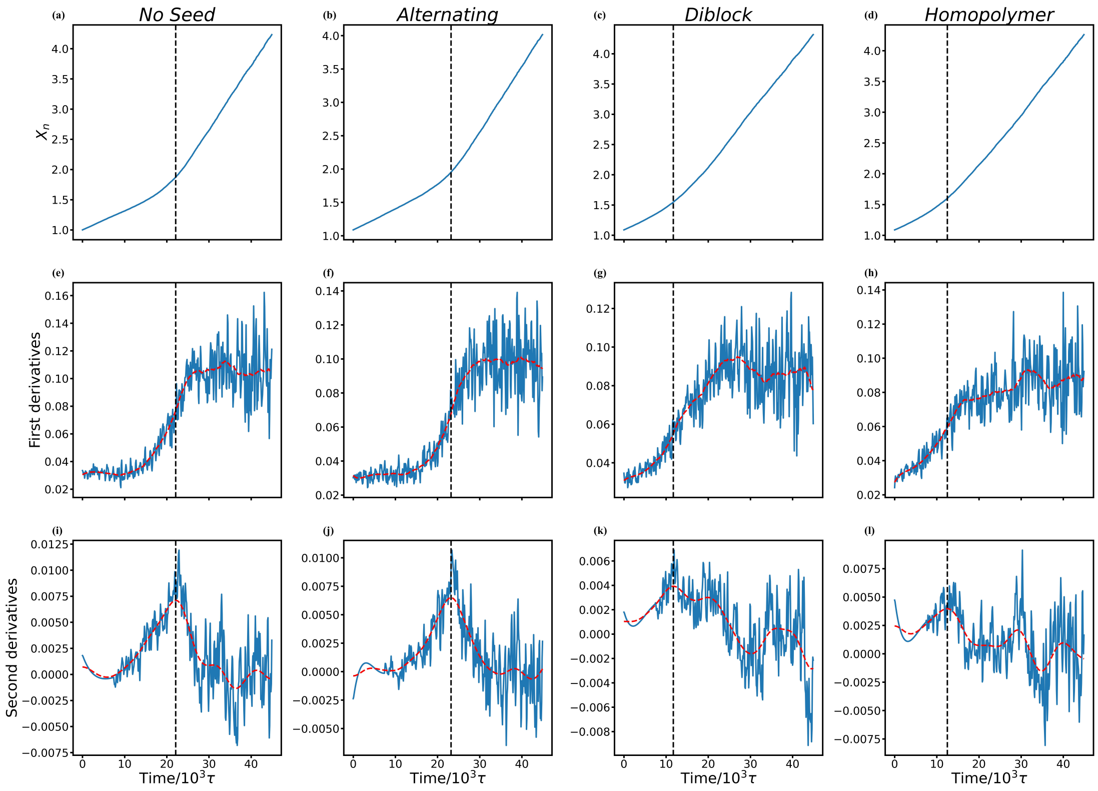

Figure S3: Turning point determination for simulations with no seed (left column) or with twenty 30-mer seeds (right three columns) with the following seed sequences: alternating (second column from the left), diblock (third column), and homopolymer (fourth column). The figures in the first row (a-d) are the X_n curves from the simulation data over the range of 0 to 45k τ. The blue lines in the second row (e-h) are the gradients of the X_n curves, and the red dashed lines are the smoothed data from a Saviztky-Golay filter with a window length of 15.5k τ and an order of 5. The blue solid lines in the third row (i-l) are the gradients of smoothed first derivatives, the red lines are the smoothed data from Gaussian filter with a sigma of 1.2k τ. The black dashed lines are the identified turning points from finding the maximum of the smoothed second derivatives (red dashed lines in (i-l)).

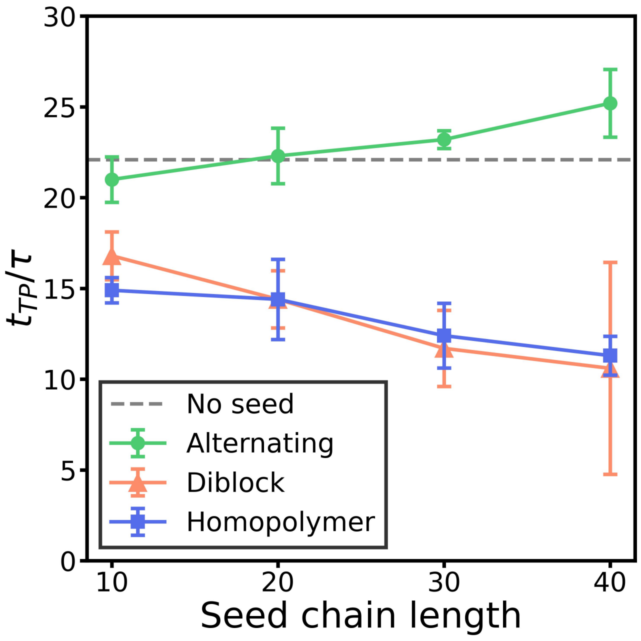

Figure S4: Turning points for varying seed lengths with different sequences, as obtained from the degree of polymerization plots (Figure S3). The results from simulations with no seeds are also included for comparison. The error bars are calculated from three independent simulations.

Chain distribution. The chain distributions with no seeds and with seeds of 30 monomers are exhibited in Figure S5. The chain length distribution of diblock seeds is broader than the other three, which agrees with a larger dispersity value at the end of reactions.

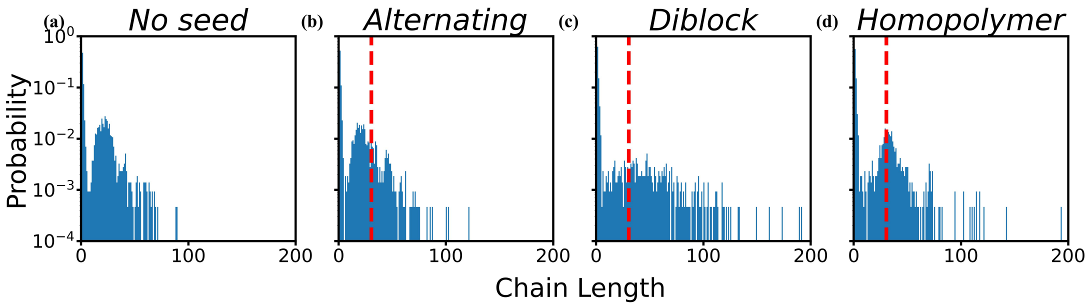

Figure S5: Chain length distributions from simulations (a) with no seed or with (b-d) twenty 30mer seeds. The sequence of seeds is (b)alternating, (c) diblock or (d) homopolymer. These chain length distributions are plotted when the reaction extent is 0.9. The red dashed line represents the chain length of seed oligomers which is 30 for these simulations.

Block degree of polymerization. The block degree of polymerization, block X_n, reports on the average length of the contiguous all-A or all-B blocks. Block X_n is related to the mixing free energy of polymer chains and monomers in implicit solvent. The development of the block X_n value vs. reaction time is shown in Figure S6(a). Direct comparisons are made for the different seeding cases in panel (b), which shows the block X_n values at $t = 0$ and at the turning point time (see inset in panel (a), where the values of the block X_n at the turning points determined in Figure S3 can be more clearly seen).

The addition of diblock and homopolymer seeds increases the initial block X_n value at $t = 0$, while also decreasing the block X_n value at the turning point. As a result, the gap between the initial block X_n and the block X_n at turning point decreases in the presence of these seeds.

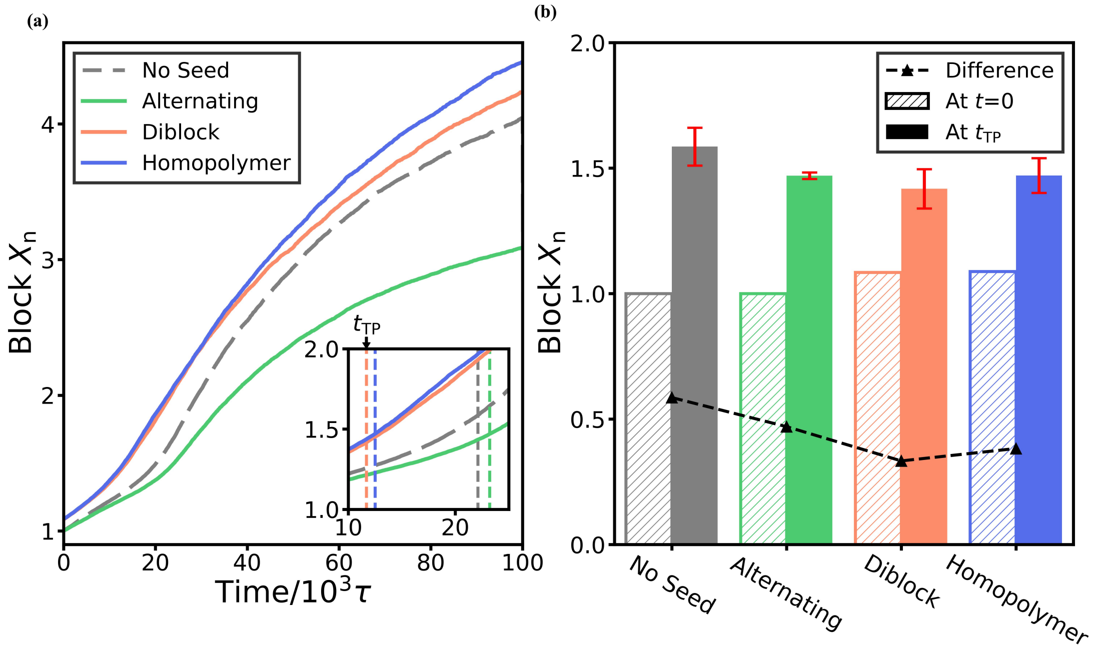

Figure S6: (a) Block degree of polymerization, block X_n, for simulations with twenty 30mer seeds compared to simulations without seeds. The inset figure shows the change of block X_n at the early stage, and the dashed lines represent the turning points.(b) Block X_n at t=0 and at the identified turning points. The red lines are the error bars from the three independent simulations for each condition. (The value at $t = 0$ is defined by the initial number and length of the blocks in the pre-formed seeds and does not vary across independent runs.) The dashed line with triangle markers represent the difference between the block X_n values at t=0 and at the turning points.

S3 The effect of seeding depends on seed length and chain flexibility.

Probability of neighbor pairs. The probability of finding like neighboring pairs in the sequence, $p_{\mathrm{AA,BB}}$, is shown in Figure S7 vs. seed length. $p_{\mathrm{AA,BB}}$ is calculated from the sum of p_{AA} and p_{BB}.[1] The pairs in the seed oligomers are excluded from the calculation.

The addition of alternating seeds generates a similar $p_{\mathrm{AA,BB}}$ compared with the no seed simulations for the shorter seed lengths. However, as the seed length increases, the $p_{\mathrm{AA,BB}}$ decreases, indicating the effect of alternating seeds may strengthen as the seeds lengthen. In contrast, the addition of even shorter diblock or homopolymer seeds results in a higher $p_{\mathrm{AA,BB}}$ compared with no seed simulations. The lengthening of the diblock seeds causes an increase in $p_{\mathrm{AA,BB}}$, indicating a tendency towards like neighbors and longer blocks with longer seed oligomers.

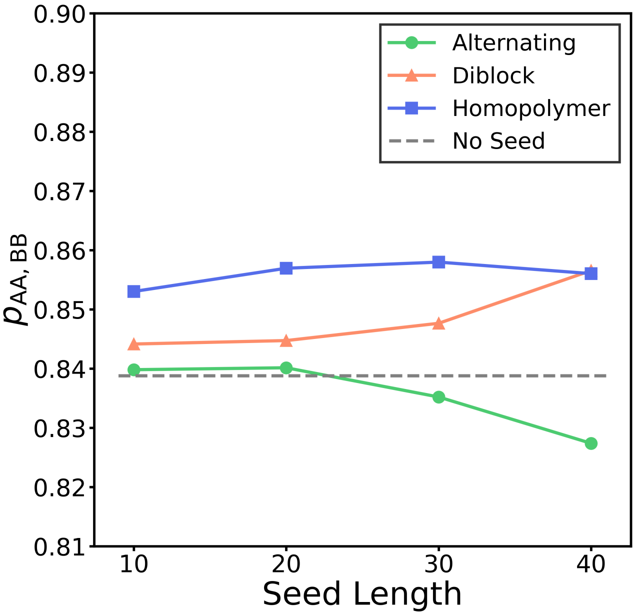

Figure S7: Probabilities of finding like neighbors, $p_{\mathrm{AA,BB}}$ for simulations with seeds compared with the ones without seeds. The length of seeds varies from 10 to 40 monomers, and the number of seeds is twenty. The pairs within the seeds are excluded from the calculation of $p_{\mathrm{AA,BB}}$.

Characteristic length. The characteristic length refers to the length with the highest probability to appear. After the simulations, we find the characteristic length via identifying local maxima with the highest probability for blocks longer than two monomers in length. To more accurately identify the maximum, we first smooth the distributions with Gaussian fitting, using a sigma of 1.2 monomers. The results of the fitting and peak finding procedures for simulations with varying seed chain lengths and without seeds are shown in Figure S8. All identified characteristic block lengths are collected in Figure S8, panel n for further analysis. Here we see that, for alternating seeds, the varying chain length does not affect the characteristic length which is approximately the characteristic length with no seed (dashed grey line). However, diblock seeds regulate the resulting chain sequence when the seed block length is over 10 monomers, as demonstrated by final characteristic block lengths that are similar to those of the seed blocks (indicated by the dashed black line). Finally, the characteristic block length of simulations with homopolymer seeds remains close to the seed block length when the seeds are 20 and 30 monomers in length, although the peak is reduced and harder to distinguish in a broader distribution. By 40 monomers in length, the distribution breadth in the homopolymer seed case makes it difficult to identify a peak, leading to significant differences between the characteristic block length identified with our criteria and the seed block lengths (see panel l).

We apply the same distribution smoothing and peak finding strategies to the block length distributions from simulations with diblock seeds of various chain stiffnesses in Figure S9, and Figure S10 shows the comparison between the identified characteristic lengths with the original seed block lengths.

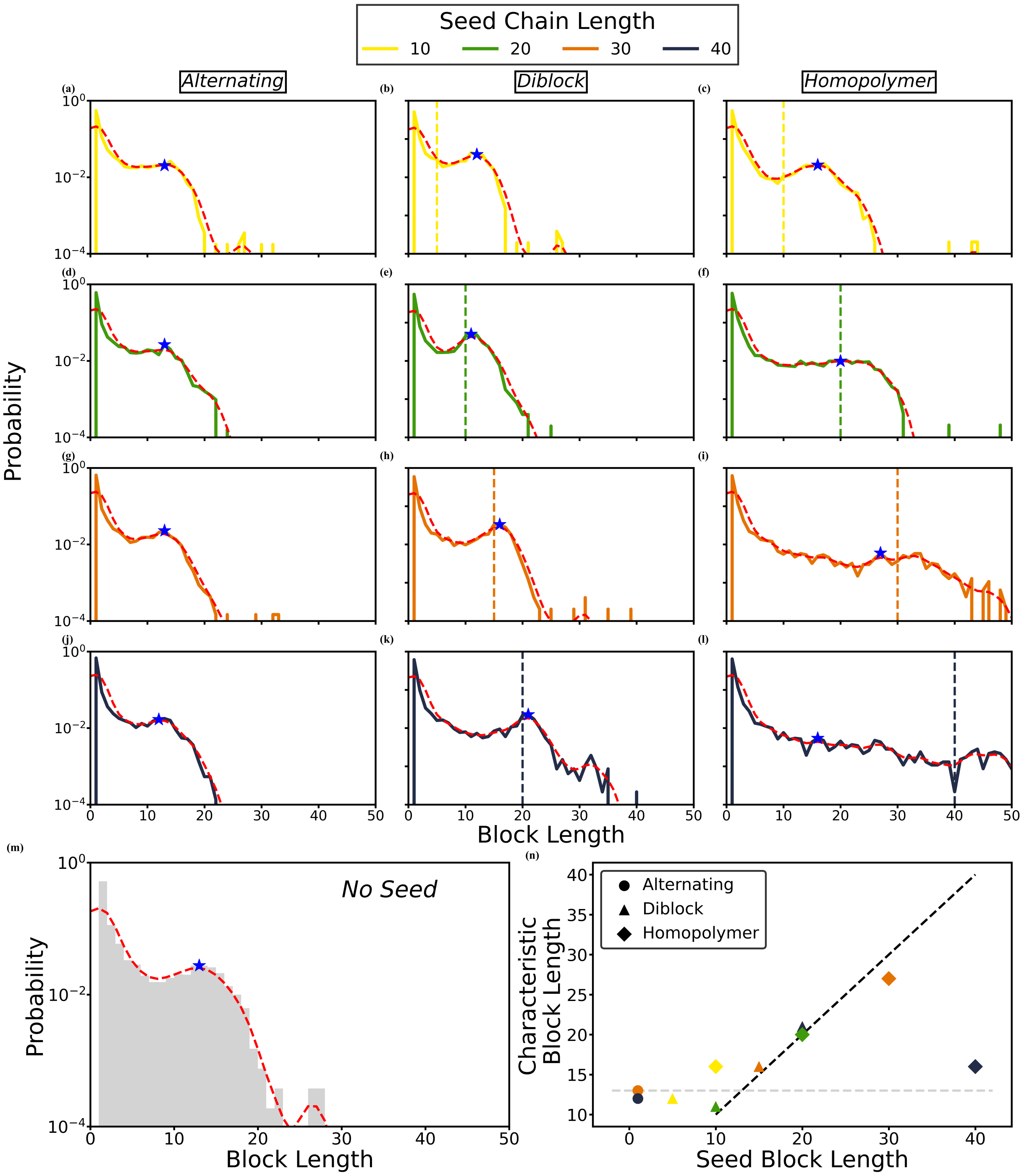

Figure S8: Identifying characteristic lengths for simulations (a-l) with varying seed chain lengths or (m) without seeds. The solid lines (a-l) or the grey shade (m) show block length distributions from the simulations, and the red dashed lines represent the Gaussian fitting of distributions with a sigma of 1.2 monomers. The blue stars mark the peaks identified after fitting by finding the local maxima with the highest probability for any block length longer than 2 monomers. The seed block lengths are indicated with dashed vertical lines (except for alternating seeds, where the seed block length is one). (n) Characteristic block lengths are compared with seed block lengths. The grey dashed horizontal line is the characteristic block length of the simulation without seeds from (m). The black dashed line shows the case where the characteristic block length is the same as the seed block length.

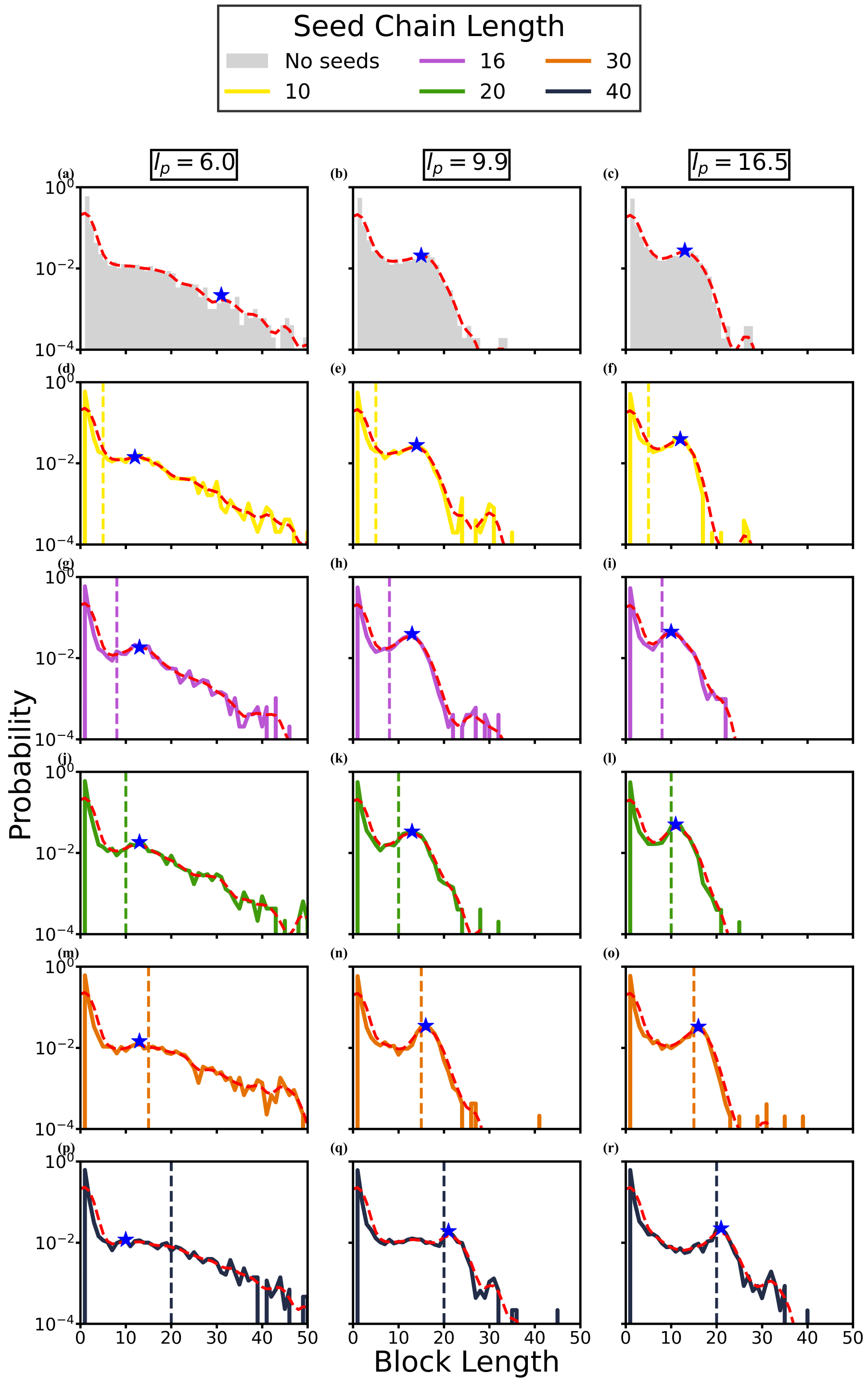

Figure S9: Identifying characteristic lengths for simulations with diblock seeds of various chain stiffnesses and seed lengths. The solid lines show the block length distributions from seeded simulations, while the red dashed lines represent the Gaussian fitting of distributions with a sigma of 1.3 monomers. The blue stars mark out the peaks identified after fitting by finding the local maxima with a block length longer than 2 and the highest probability. To enable comparison with the seed sequences, the seed block lengths are indicated with dashed vertical lines.

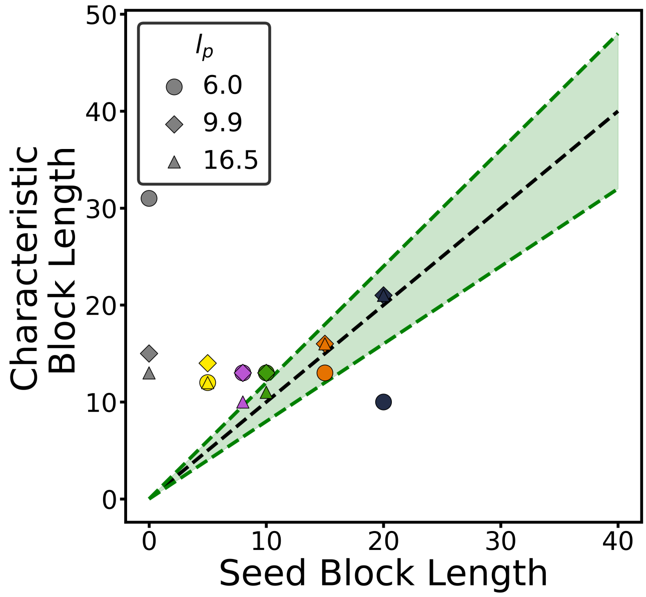

Figure S10: Characteristic lengths for simulations with different seed chain stiffness from Figure S9. The shape of data points is related to the chain stiffness, while the color is related to the seed chain length as shown in Figure S9. The black dashed line represents the case where characteristic length is the same as seed block length. The green shade between the two green dashed lines represents the range within ±20% of the seed block lengths.

Order parameter and snapshots for simulations with varying chain stiffness. Figure S11 shows the local order parameters for simulations with diblock seeds of varying chain stiffness and length. The local nematic order parameter of flexible chains in Figure S11(a), with a persistence length of $l_p = 6.0$ monomers, did not increase as significantly as with the stiffer chains in Figure S11(b,c), with persistence lengths of l_p=9.9, 16.5, indicating that the nascent aggregation and alignment is less favorable for the flexible chains as compared to the stiffer chains.

Figure S12 displays representative snapshots at the end of these reactions with diblock seeds. The snapshots reinforce the findings in Figure S11, demonstrating that stiff chains form long straight bundles (Figure S12 (b-c, e-f)), while flexible seed chains curl (Figure S12(a,d)), which affects their contact interface with monomers and newly formed oligomers. The curling becomes more visible as the seed length increases (compare Figure S12(a)&(d)). Without aligned bundles of seed chains in the middle, the regulation of block length for newly formed chains from seed chains is weakened.

Both the order parameter and the snapshot provide evidence of the decreasing aggregation of seeds with decreased stiffness, explaining why the seeding effect of flexible chains is not as strong as that of stiff chains.

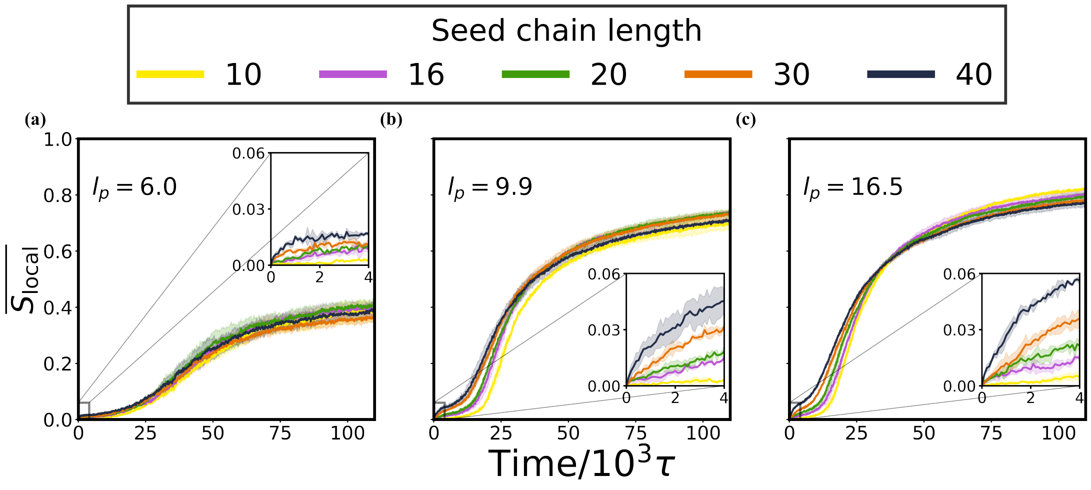

Figure S11: Local nematic order parameters vs. reaction time in the presence of diblock seeds with different persistent lengths, l_p, and seed lengths. The inset figures show the order parameter at the beginning of reaction from 0 to 4k τ. The shaded region represents the variation in $\overline{S_{\text{local}}}$ from three independent simulations run at the same conditions.

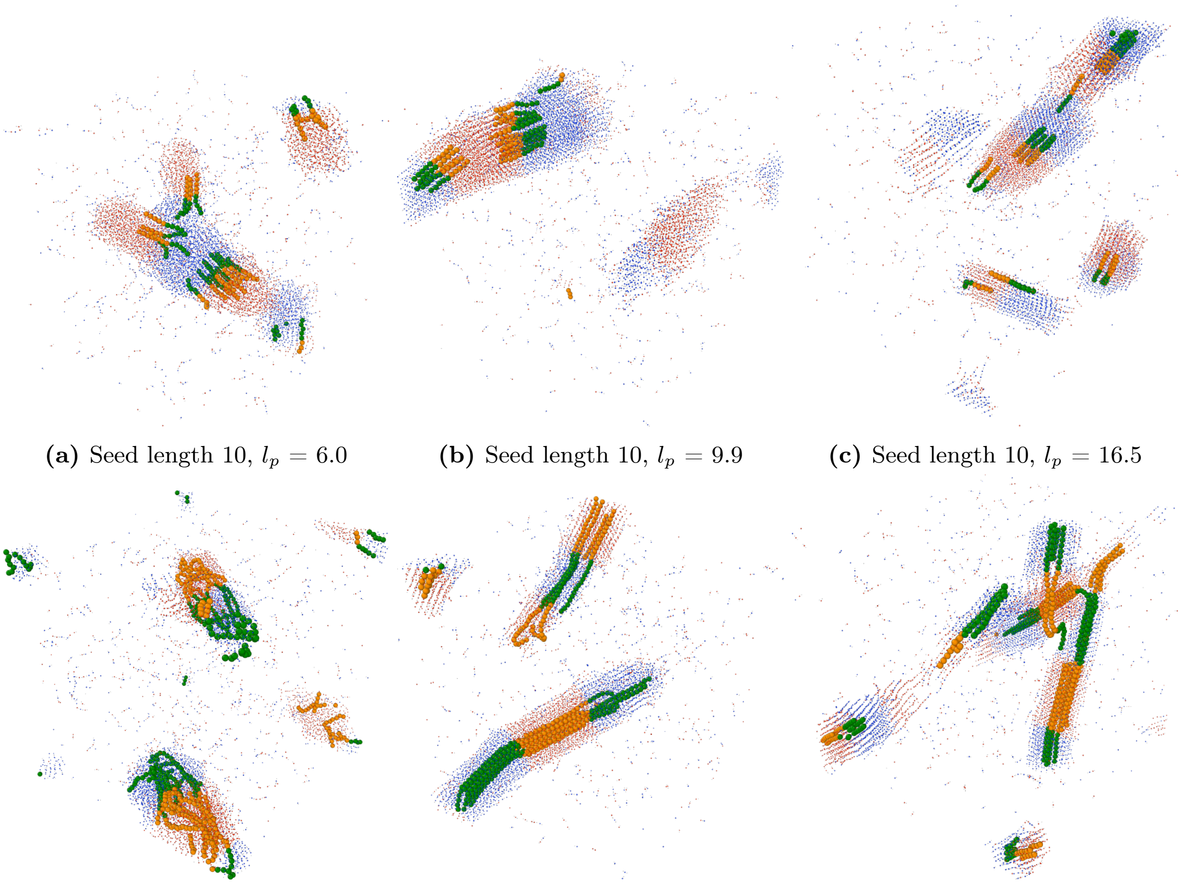

(a) Seed length 10, $l_p = 6.0$ **(b)** Seed length 10, $l_p = 9.9$ **(c)** Seed length 10, $l_p = 16.5$

(d) Seed length 30, $l_p = 6.0$ **(e)** Seed length 30, $l_p = 9.9$ **(f)** Seed length 30, $l_p = 16.5$

Figure S12: Snapshots of simulation systems with diblock seeds at $p = 0.9$. (a-c) are snapshots of systems with 10-monomer diblock seeds. (d-f) are snapshots of systems with 30-monomer diblock seeds. The snapshots in the same column refers to the systems with the same chain stiffness. From left to the right, the persistent lengths are 6.0, 9.9, and 16.5 monomers. The free monomers at the beginning of the reaction are represented by red and blue particles for monomer **A** and **B**, while the orange and green particles show the **A** and **B** monomers in the seed chains, the size of which are artificially enlarged in this visualization to highlight the locations of the seeds within the final structures.

Simulations with asymmetric persistence length. We run further simulations on the addition of seed oligomers with monomers of different persistence lengths, and the results are shown in Figure S13. Here the persistence length of the A monomer is 16.5 monomers, while the persistence of the B monomer is 6.0 monomers. We add twenty diblock seeds with chain lengths of 30-monomers each into the simulation, in which the length of the A and B blocks is either 10 monomers (Figure S13(b) and (e)) or 15 monomers (Figure S13(c) and (f)). The stiffer A-block results in an obvious characteristic block length at around the seed block length for both long and short blocks. The more flexible B-block is broader than A-block.

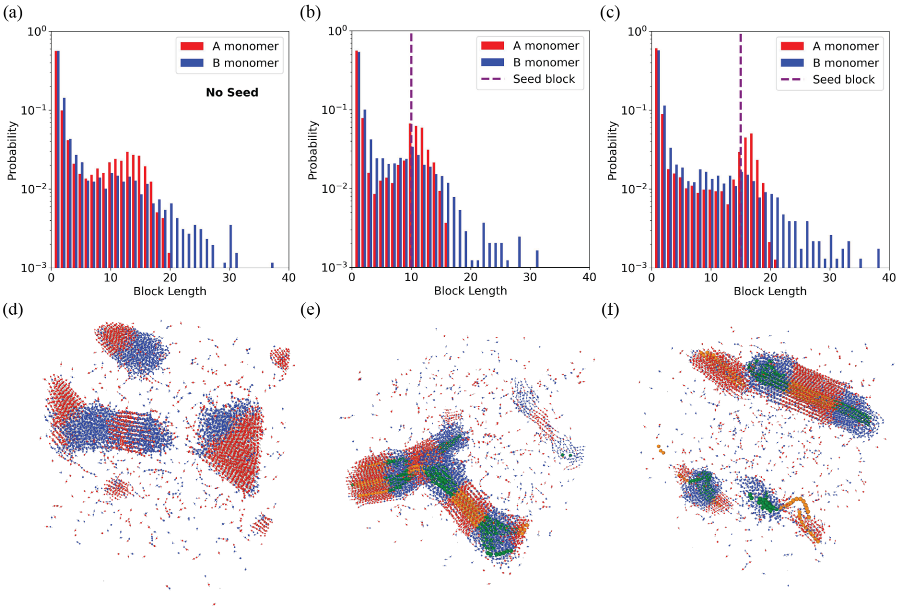

Figure S13: Block length distribution of A (red) and B (blue) monomers and final snapshots for the simulations of rod-coil polymers with different persistence lengths for A and B. Panel (a) and (d) are from simulations without seeds for comparison. The simulations with seeds contain twenty seeds with a length of 30-monomers. The block lengths of A and B are either 10 monomers (panel (b) and (e)) or 15-monomers (panel(c) and (f)), and are represented by the purple dashed lines in the block length distributions. The persistence length of A is 16.5 monomers, while that of B is 6.0 monomers.

Snapshots for simulations with varying seed block lengths. Representative snapshots at the end of the reactions are displayed in Figure S14 for simulations examining the effect of seed block length to provide insights for the resulting block length distributions. When the seed block length is below 5 monomers (Figure S14a&b), the seeds are located on the outside of the chain aggregates. Once the seed block length reaches 10 monomers, the seeds are found within the core of the final chain aggregates (Figure S14c-e). When the seed block length is 20 monomers, then the seeds are homopolymers and aggregates that are entirely A or B can be found. It is also interesting to note that, given the persistence length of 9.9 monomers, at twenty monomers, some seeds begin to fold on themselves, as can be seen in the upper right hand corner of panel (e).

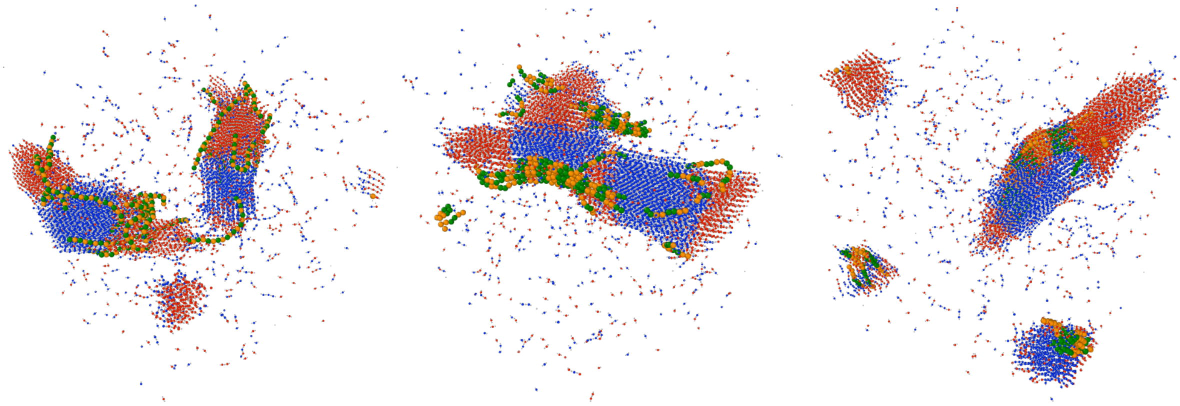

(a) Seed block length: 1 **(b)** Seed block length: 2 **(c)** Seed block length: 5

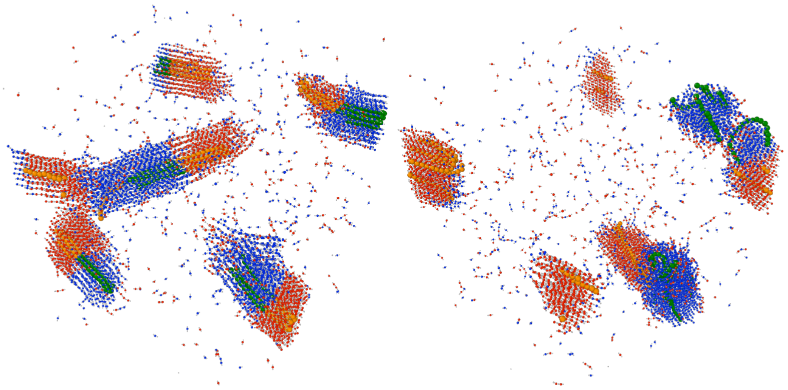

(d) Seed block length: 10 **(e)** Seed block length: 20

Figure S14: Snapshots at $p = 0.9$ for the simulations investigating the effect of seed block lengths. The persistence length for the chains in all simulations is 9.9 monomers. The free monomers at the beginning of the reaction are represented by red and blue particles for monomer **A** and **B**, respectively, while the orange and green particles indicate the **A** and **B** monomers of the seed chains, the size of which are artificially enlarged to highlight their locations.

S4 The effect of seeding mitigates the effect of diffusion and reaction timescales on block-length distributions.

Relative kinetic timescale calculation. The relative kinetic timescale is the ratio between diffusion timescale, τ_{D}, and reactive timescale, τ_{R}. We take the definition of τ_{R} as $\tau_{\mathrm{R}} \equiv k_{\mathrm{eff}}^{-1}$,[9] in which the effective kinetic rate constant, k_{eff}, is related with the activation energy, E_a in the equation:

$$k_{\mathrm{eff}} = (A_{\mathrm{AA,BB}} + A_{\mathrm{AB}}) \cdot \exp(-\beta E_{\mathrm{a}}), \tag{S10}$$

which is derived in our previous work[4] at a symmetric limit where interactions between all pairs of like monomers are the same. A_{ij} is the Arrhenius constant for bond formation, and the 'AA,BB' notation refers to the bonding between like neighbors, **AA** or **BB**, while the 'AB' notation refers to unlike neighbors, **AB**. According to our previous work,[4] $A_{\mathrm{AA,BB}}$, is about 1.4 when $\varepsilon_{\mathrm{AA,BB}}$ is 1 $k_{\mathrm{B}}T$, and A_{AB} is about 0.6 when $\varepsilon_{\mathrm{AB}}$ is 0 $k_{\mathrm{B}}T$. The activation energy, E_{a}, is the same for bond formation between all types of monomers, and arises from a combination of the LJ steric repulsions between central particles, the geometric constraints of intramonomer bondings, and the short-range repulsion between linking particles. The variation in activation energies in this work results from our adjustment of the strength of the short-range repulsion between linking particles.

The diffusion timescale, τ_{D}, is defined as the time required for a particle to diffuse its own size, as defined by the particle diameter, σ. It depends on the viscosity, η, in a formula of:

$$\tau_{\mathrm{D}} \equiv \frac{\sigma^2}{D} = \beta\eta\sigma^2, \tag{S11}$$

where D is the diffusion coefficient. Since in Langevin dynamics, D is related to viscosity, η, according to $\eta = \frac{k_B T}{D}$, we can calculate τ_{D} using Equation S11.

The relative kinetic timescales are summarized in Table S5.

Table S5: Relative kinetic timescales used in this paper with varying viscosity or activation energy, E_a.

Viscosity/mPa · s	$E_a/k_{\mathrm{B}}T$	$\log(\tau_{\mathrm{D}}/\tau_{\mathrm{R}})$
0.0091	8.3	-7.6
0.0182	8.3	-6.9
0.0910	8.3	-5.3
0.9100	8.3	-3.0
0.0910	6.8	-3.8
0.0910	9.8	-6.9

Block length distributions of simulations with varying viscosity and activation energy. The block length distributions for simulations with varying viscosity or varying activation energy are shown in Figure S15. The simulations are either with no seed or with twenty diblock seeds of varying length. The identification of characteristic lengths is the same as detailed in Section **S3** with a sigma of 0.8 monomer for Gaussian fitting considering the width of peaks. The fitting and peak finding results are shown in Figure S16, and the characteristic block lengths obtained are then included in Figure 7 in the main paper.

Wasserstein distance calculation. The Wasserstein distance is a statistical metric describing the difference between two probability distributions.[10] The first-order Wasserstein distance is calculated from:

$$W_1(p(x), q(x)) = \int_{-\infty}^{\infty} |P(x) - Q(x)|, \tag{S12}$$

where $p(x)$ and $q(x)$ are the two probability distributions, and $P(x)$ and $Q(x)$ are their respective cumulative distribution functions.

Here we use the Wasserstein distance to compare the block length distributions obtained from simulations with the ideal distributions obtained from Markovian statistics, which is calculated as:

$$P(n) = (1 - p \cdot p_{\mathrm{AA,BB}})(p \cdot p_{\mathrm{AA,BB}})^{(n-1)}, \tag{S13}$$

where $P(n)$ is the probability of finding a block with a length of n monomers, p is the reaction extent, and $p_{\mathrm{AA,BB}}$ is the probability of finding the same neighbors which is either **AA** or **BB**. In our previous work,[4] we derived the theoretical $p_{\mathrm{AA,BB}}$ values for different values of $\varepsilon_{\mathrm{AA,BB}}$ and $\varepsilon_{\mathrm{AB}}$, in the absence of any emergent phase separation, based on the kinetic parameters from Mayo-Lewis theory.[11] Since the activation energy for bond formation is the same for the **AA**, **AB**, and **BB** pairs within in this work, the equation can be simplified to:

$$p_{\mathrm{AA,BB}} = \frac{\frac{A_{\mathrm{AA,BB}}(\epsilon_{\mathrm{AA,BB}})}{A_{\mathrm{AB}}(\epsilon_{\mathrm{AB}})}}{\frac{A_{\mathrm{AA,BB}}(\epsilon_{\mathrm{AA,BB}})}{A_{\mathrm{AB}}(\epsilon_{\mathrm{AB}})} + 1}. \tag{S14}$$

The parameters here are explained in Equation S10. The calculated Wasserstein distance results are shown in Figure 7 in the main paper.

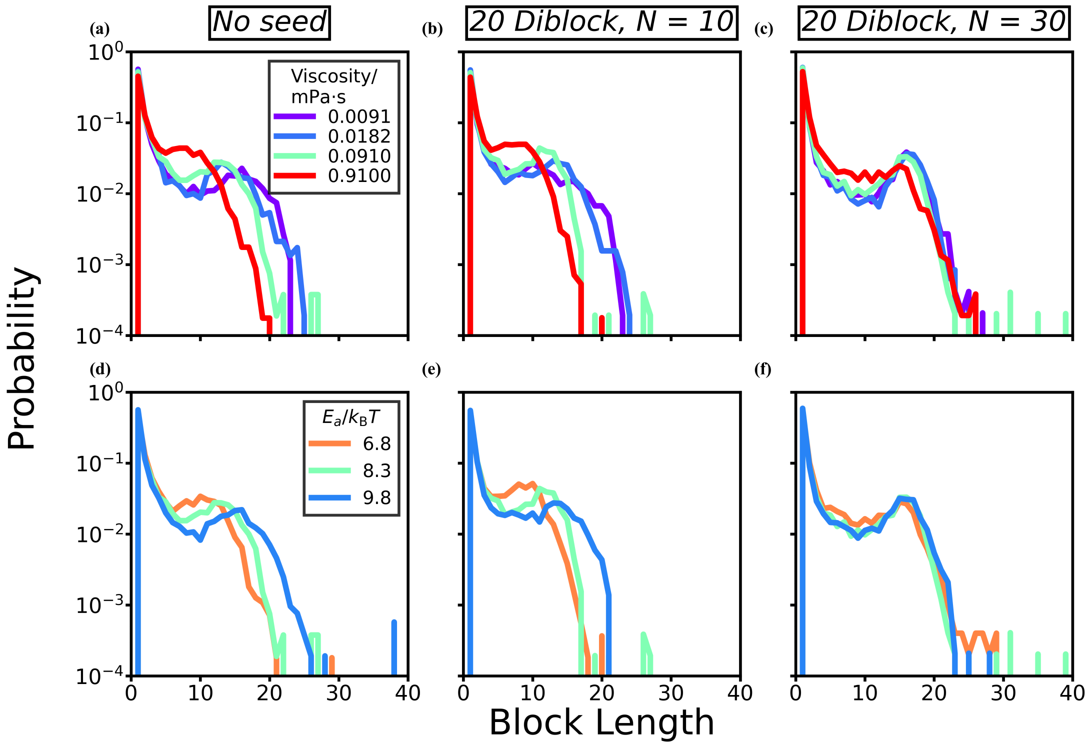

Figure S15: Block length distributions across varying viscosities (a-c) and varying activation energies (d-f). The results with no seeds are shown in (a) and (d), the results with twenty short, 10-monomer, diblock seeds are shown in (b) and (e), and the results with twenty longer, 30-monomer, diblock seeds are shown in (c) and (f).

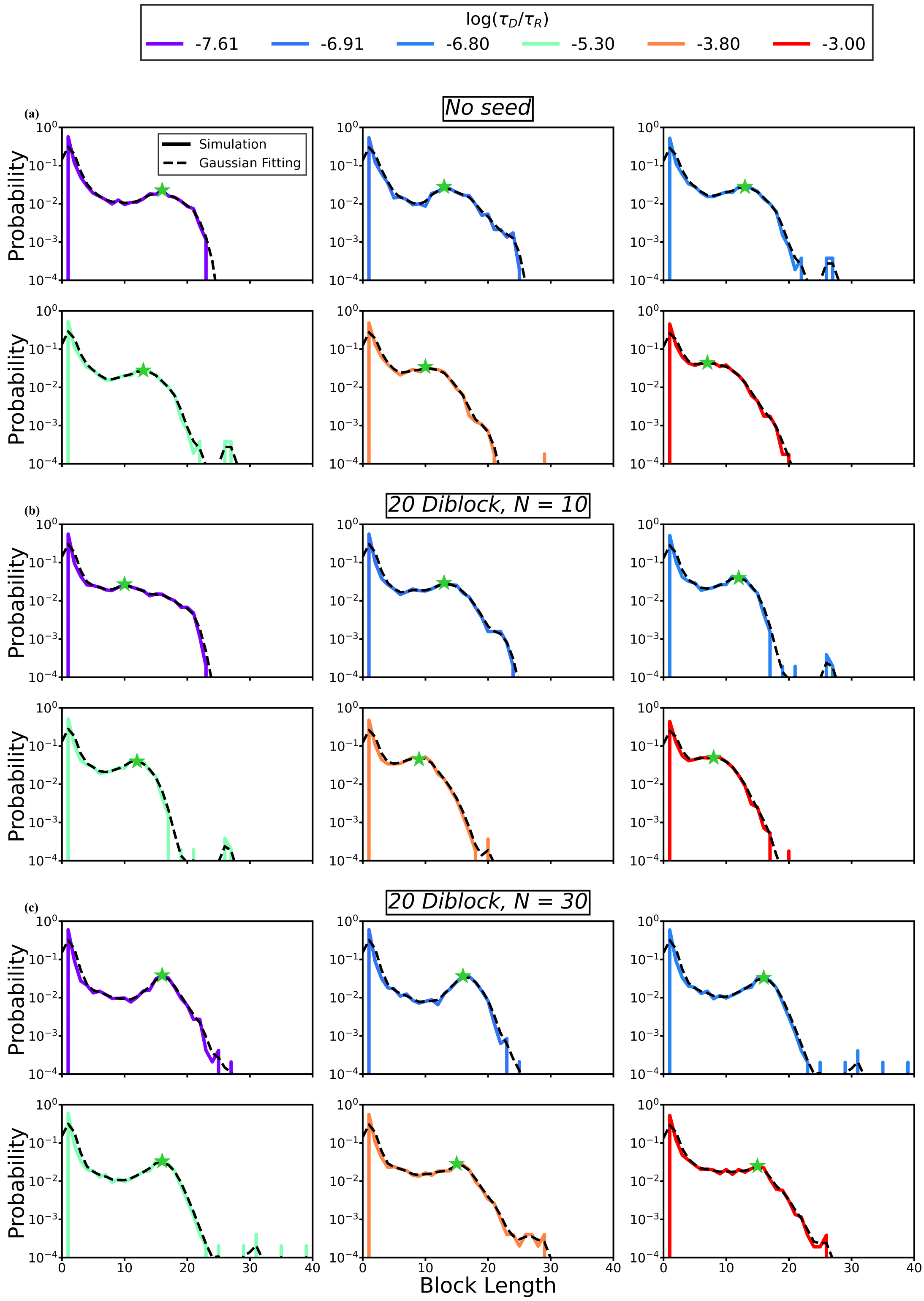

Figure S16: Identifying characteristic block lengths for simulations (a) without seeds and with twenty diblock seeds with a length of (b) 10 monomers and (c) 30 monomers. The solid lines show the block length distributions from seeded simulations, while the black dashed lines represent the Gaussian fitting of distributions with a sigma of 0.8 monomers. The green stars are the characteristic lengths identified after fitting by finding the highest local maxima with a block length longer than two monomers.

References

(1) Zhang, Z.; DuBay, K. H. Modeling the Influence of Emergent and Self-Limiting Phase Separations among Nascent Oligomers on Polymer Sequences Formed during Irreversible Step-Growth Copolymerizations. Macromolecules **2019**, *52*, Publisher: ACS Publications, 5480–5490.

(2) Zhang, Z.; DuBay, K. H. The Sequence of a Step-Growth Copolymer Can Be Influenced by Its Own Persistence Length. The Journal of Physical Chemistry B **2021**, *125*, 3426–3437.

(3) Thompson, A. P.; Aktulga, H. M.; Berger, R.; Bolintineanu, D. S.; Brown, W. M.; Crozier, P. S.; in 't Veld, P. J.; Kohlmeyer, A.; Moore, S. G.; Nguyen, T. D.; Shan, R.; Stevens, M. J.; Tranchida, J.; Trott, C.; Plimpton, S. J. LAMMPS - a flexible simulation tool for particle-based materials modeling at the atomic, meso, and continuum scales. Computer Physics Communications **2022**, *271*, 108171.

(4) Nguyen, N. Q.; Hamblin, R. L.; DuBay, K. H. Emergent Sequence Biasing in Step-Growth Copolymerization: Influence of Non-Bonded Interactions and Comonomer Reactivities. The Journal of Physical Chemistry B **2022**, *126*, 6585–6597.

(5) Peng, C.-K.; Buldyrev, S. V.; Goldberger, A. L.; Havlin, S.; Sciortino, F.; Simons, M.; Stanley, H. E. Long-range correlations in nucleotide sequences. Nature **1992**, *356*, 168–170.

(6) Rumyantsev, A. M.; Johner, A.; de Pablo, J. J. Sequence Blockiness Controls the Structure of Polyampholyte Necklaces. ACS Macro Letters **2021**, *10*, 1048–1054.

(7) Rumyantsev, A. M.; Jackson, N. E.; Johner, A.; de Pablo, J. J. Scaling Theory of Neutral Sequence-Specific Polyampholytes. Macromolecules **2021**, *54*, 3232–3246.

(8) Savitzky, A.; Golay, M. J. E. Smoothing and Differentiation of Data by Simplified Least Squares Procedures. Analytical Chemistry **1964**, *36*, 1627–1639.

(9) Eva-Maria Wartha, M. B.; Harasek, M. Characteristic Chemical Time Scales for Reactive Flow Modeling. Combustion Science and Technology **2021**, *193*, 2807–2832.

(10) Panaretos, V. M.; Zemel, Y. Statistical Aspects of Wasserstein Distances. Annual Review of Statistics and Its Applicati **2019**, *6*, 405–431.

(11) Mayo, F. R.; Lewis, F. M. Copolymerization. I. A Basis for Comparing the Behavior of Monomers in Copolymerization; The Copolymerization of Styrene and Methyl Methacrylate. Journal of the American Chemical Soci **1944**, *66*, 1594–1601.
